# Acute kidney injury after myocardial infarction: prognostic implications via dual robust methods

**DOI:** 10.3389/fmed.2025.1555478

**Published:** 2025-07-22

**Authors:** Pan Guo, Fang Tao, Lili Du, Hongmei Yang, Wenguang Wang, Chunpeng Ma, Xile Bi, Lin Ren, Hongtao Yin, Lixiang Ma

**Affiliations:** ^1^Department of Cardiology, Qinhuangdao First Hospital, Qinhuangdao, Hebei, China; ^2^Medical Department, Qinhuangdao First Hospital, Qinhuangdao, Hebei, China; ^3^Department of Cardiology, Tianjin Medical University General Hospital, Tianjin, China

**Keywords:** myocardial infarction, acute kidney injury, prognosis, propensity score matching, doubly robust analysis

## Abstract

**Background:**

Acute kidney injury (AKI) following acute myocardial infarction (AMI) notably affects patient outcomes. The impact of KDIGO AKI staging on post-discharge short- and long-term outcomes, particularly early-stage AKI, is not well understood. This study evaluates the prognostic implications of various KDIGO stages in AMI patients.

**Methods:**

Utilizing the Medical Information Mart for Intensive Care IV (version 3.0) database, this retrospective cohort study included adult patients primarily diagnosed with AMI. Statistical analyses, including doubly robust estimation, propensity score matching, logistic regression, and Cox regression, were performed. The study compared Non-AKI (KDIGO stage 0) with Mild-AKI (maximum KDIGO stage 1 during hospitalization), and Normal-or-mild AKI (KDIGO stages 0–1) with Moderate-to-severe AKI (KDIGO stages 2–3).

**Results:**

Among 5,715 patients analyzed, 4,306 (75.36%) developed AKI. Doubly robust analysis revealed no significant differences in outcomes between Non-AKI and Mild-AKI groups (28-day mortality: OR 0.97, 95% CI 0.68–1.38; 180-day mortality: HR 0.94, 95% CI 0.76–1.18; 1-year mortality: HR 0.98, 95% CI 0.81–1.20). However, Moderate-to-severe AKI was significantly associated with worse outcomes compared to Normal-or-mild AKI (28-day mortality: OR 1.67, 95% CI 1.36–2.05; 180-day mortality: HR 1.06, 95% CI 1.02–1.10; 1-year mortality: HR 1.22, 95% CI 1.07–1.38; all *p* < 0.001). Subgroup analyses revealed that patients under 65 years with Mild-AKI showed higher risks of 180-day and 1-year mortality compared to Non-AKI, while Moderate-to-severe AKI consistently demonstrated worse outcomes across all subgroups (age, SOFA score, heart failure status, and renal disease status). These findings were robust across multiple sensitivity analyses.

**Conclusions:**

Patients with Mild-AKI can be considered as having “subclinical AKI,” with prognoses similar to Non-AKI patients. In contrast, Moderate-to-severe AKI significantly worsens prognosis compared to Normal-or-mild AKI.

## Introduction

In recent years, growing attention has been directed toward the interplay between cardiovascular conditions, including acute heart failure, acute myocardial infarction (AMI), and cardiovascular surgery, and the onset of acute kidney injury (AKI). This is due to the profound impact AKI has on patient outcomes and prognosis (1). Research has suggested a strong correlation between cardiac and renal function, giving rise to the term “cardiorenal syndrome (CRS)” in the context of heart failure and acute coronary syndrome (ACS) (2).

The pathophysiology of CRS is characterized by a complex interplay of hemodynamic and non-hemodynamic factors that cause mutual cardiac and renal damage. Key contributors include common risk factors such as hypertension, diabetes, atherosclerosis, and chronic inflammation, which drive disease progression (3). Hemodynamic disturbances like venous congestion and increased intra-abdominal pressure reduce renal blood flow, impair glomerular filtration, and activate the renin-angiotensin system (RAAS), worsening renal function (4, 5). Non-hemodynamic mechanisms involve neurohormonal dysregulation, oxidative stress, and inflammation, which contribute to chronic renal hypoxia, tissue injury, and fibrosis (6–8). Inflammatory mediators such as TNF-α, IL-1, and IL-6 play crucial roles, leading to both cardiac and renal remodeling (9). Endothelial dysfunction further exacerbates the cycle of damage by impairing vasodilation, increasing vascular permeability, and promoting thrombosis and atherosclerosis (10). Together, these mechanisms create a self-perpetuating cycle of organ dysfunction, contributing to the progression of CRS (2).

The occurrence of AKI following AMI significantly prolongs hospital stay, increases medical costs, and elevates both short- and long-term mortality. Reported incidence rates of AKI after AMI range from 5.2% to 59% across studies, primarily due to variations in the criteria used to define AKI and differences in study populations (11).

Currently, the most widely used criteria for diagnosing AKI include the RIFLE criteria (12), AKIN criteria (a later version of the RIFLE classification) (13), and KDIGO guidelines (14). Researchers applied both the KDIGO and RIFLE criteria to AMI patients, revealing that KDIGO detects AKI more effectively than RIFLE, with detection rates of 36.6% vs. 14.8% (15). KDIGO integrates elements from both RIFLE and AKIN, combining their strengths to offer a more standardized and comprehensive definition of AKI, thereby minimizing discrepancies between diagnostic frameworks (11). KDIGO is notably more sensitive in detecting AKI, particularly in the early stages (16). Its three-stage classification simplifies clinical application while preserving the diagnostic accuracy of more complex systems like RIFLE. Although AKI diagnosis relies on acute increases in serum creatinine (SC) and reduced urine output (UO), UO measurement is underutilized in clinical practice, despite evidence suggesting its significant diagnostic and prognostic value (17). The use of the more sensitive KDIGO criteria, which incorporate UO, may thus provide an advantage in detecting AKI in patients with AMI. However, few studies have examined the impact of KDIGO staging on short- and long-term outcomes in AMI patients after discharge, and it remains unclear whether even early-stage AKI affects prognosis. Therefore, this study aims to comprehensively analyze outcomes in AMI patients across different KDIGO AKI stages to reveal the prognostic implications of each stage.

## Methods

### Study design

This study conforms to the Strengthening the Reporting of Observational Studies in Epidemiology (STROBE) guidelines, as outlined in the Supplementary materials. It aims to investigate the short-term and long-term impacts of mild and moderate-to-severe AKI on the prognosis of ICU patients with acute myocardial infarction, utilizing real-time monitoring of AKI based on KDIGO criteria. The KDIGO criteria model in MIMIC-IV dynamically evaluated AKI stages through serum creatinine changes over the past seven days and 48 h, alongside hourly urine output monitored over 6, 12, and 24-h intervals. This approach improved the sensitivity of AKI assessment, facilitating earlier detection and more precise classification of kidney injury. The project received approval from the institutional review boards at both the Massachusetts Institute of Technology (MIT) and Beth Israel Deaconess Medical Center (BIDMC), with informed consent being waived.

This retrospective observational study utilized data from the Medical Information Mart for Intensive Care IV (MIMIC-IV, version 3.0) database. This updated version of MIMIC-III includes critical care information for ICU patients at BIDMC from 2008 to 2022. The database contains comprehensive records from patient hospitalizations, such as laboratory tests, medications given, vital signs, and other details. Author PG gained access to the database after fulfilling the data usage agreement and obtaining Collaborative Institutional Training Initiative (CITI) certification. Since all patient information is de-identified, informed consent was not necessary (17).

### Study population

Inclusion criteria: (1) patients aged 18 years or older; (2) AMI listed among the top three discharge diagnoses. Exclusion criteria: (1) not a first hospitalization; (2) absence of ICU records; (3) ICU stay time < 1 day.

The study is divided into two parts. In the first part, patients classified under KDIGO AKI stages 0 and 1 were grouped as Non-AKI and Mild-AKI. In the second part, all patients were included, with those in KDIGO AKI stages 0 and 1 categorized as Normal-or-mild-AKI, while those in stages 2 and 3 were classified as Moderate-to-severe-AKI.

### Data extraction and preprocessing

Data extraction was performed using PostgreSQL 14 and SQL queries (Berkeley, California, USA). The dataset extracted included demographics, ICU length of stay, complications, laboratory test results, treatments, and other pertinent clinical information. Laboratory results were taken from the first tests conducted upon ICU admission, as these initial values are available quicker and support timely patient assessment with clinical prediction models. The estimated glomerular filtration rate (eGFR) was calculated using the Cockcroft-Gault (CG) equation (18): eGFR = 175 × (standardized serum creatinine)^−1.154^ × (age)^−0.203^ × 1.212 (if Black) × 0.742 (if female).

### Endpoint

The endpoints were 28-day mortality, 180-day mortality, and 1-year all-cause mortality.

### Statistical methods

The normality of continuous variables was evaluated using the Kolmogorov-Smirnov test. Variables with a normal distribution were presented as mean ± standard deviation, whereas variables that did not follow a normal distribution were expressed as median and interquartile range (IQR) [M (P25, P75)]. The homogeneity of variances for continuous variables across groups was evaluated using Levene's test. For comparisons between two cohorts, continuous variables that followed an independent normal distribution and demonstrated homogeneity of variances were analyzed using Student's *t*-test. If these assumptions were not met, the Mann-Whitney *U* test was used to assess differences between groups. For categorical variables, Fisher's exact test was applied when the sample size was < 40. Otherwise, the Chi-square test was used to assess differences between groups. Categorical data were presented as frequencies and percentages. Multiple imputation was performed using the ‘mice' package in *R* for variables with missing data. Variables with more than 20% missing values were excluded from imputation and not included in model construction. To ensure robust imputation results, the number of imputations was set to 100.

The doubly robust estimation approach was utilized to determine the independent associations between the occurrence of AKI in patients with myocardial infarction and their prognosis. This method combines outcome modeling and propensity score weighting to provide reliable estimates, even if one of the models (outcome or propensity score) is misspecified. This method, also known as survey-weighted generalized linear models, amalgamates a multivariate regression model with a propensity score model to evaluate both the correlation and the causal influence of an exposure on an outcome (19, 20). Typically, unbiased estimation of causal effects using either a regression model or a propensity score model individually is possible only when the respective statistical model is correctly specified. In contrast, the doubly robust estimator combines these two approaches, ensuring that an unbiased effect estimate can still be obtained if at least one of the models is correctly specified.

The gradient boosted model (GBM) was applied to estimate propensity scores for AKI, with the aim of minimizing covariate imbalance between the Non-AKI and Mild-AKI groups, as well as the Normal-or-mild-AKI and Moderate-to-severe-AKI groups. GBM, a machine learning algorithm, iteratively builds and combines models into an ensemble to enhance the accuracy of response variable estimates. Its main principle involves constructing new models that are highly correlated with the negative gradient of a predefined loss function. In this study, regression trees were used as the base learners for the GBM, incorporating 39 covariates in total (21).

An inverse probability of treatment weighting (IPTW) approach was applied to construct a weighted cohort, utilizing the estimated propensity scores as weights. To evaluate the performance of the propensity score model in achieving balance between the groups, covariate imbalances were analyzed for both the unadjusted and weighted cohorts. Standardized mean differences (SMDs) were computed to measure discrepancies between the groups. Subsequently, logistic regression or Cox regression was conducted on this weighted cohort, adjusting for variables that remained unbalanced between groups in the propensity score model. This approach is referred to as a doubly robust analysis using ‘survey' package. Logistic regression analyses utilized the ‘stats' package. In our study, the survival package was employed to fit Cox proportional hazards models and assess the proportional hazards (PH) assumption. For time-dependent covariates that violated the PH assumption, appropriate transformations, such as time-dependent covariate effects or stratification, were applied. These adjustments allowed for more accurate estimation of hazard ratios and improved overall model fit. The survival package provided essential functions for testing the PH assumption (e.g., cox.zph) and incorporating time-dependent effects (e.g., coxph with time-dependent covariates).

Statistical analyses were conducted using R software (version 4.4.1; R Foundation for Statistical Computing, Vienna, Austria). All tests were two-tailed, with a significance level set at *P* < 0.05.

### Sensitivity analysis

We conducted a series of sensitivity analyses to evaluate the robustness of the study's findings and to determine how our conclusions might be influenced by using different association inference models. In these analyses, we applied additional models. For the outcome of 28-day mortality, we used a Log-rank test model, a Multivariate Several models were utilized in the analysis, including a logistic regression model adjusted for all covariates, a multivariate logistic regression model adjusted for unbalanced covariates, a survey-weighted Generalized Linear Model (GLM) incorporating IPTW and adjusted for all covariates, and a survey-weighted GLM with IPTW adjusted for unbalanced covariates. For the outcomes of 180-day and 1-year mortality, the analysis employed a log-rank test, a multivariate Cox proportional hazards model adjusted for all covariates, a multivariate Cox model adjusted for unbalanced covariates, a survey-weighted Cox model with IPTW adjusted for all covariates, and a survey-weighted Cox model with IPTW adjusted for unbalanced covariates. The effect sizes and corresponding *p*-values derived from these models were reported and compared.

## Results

### Baseline characteristics

**Figure 1:**
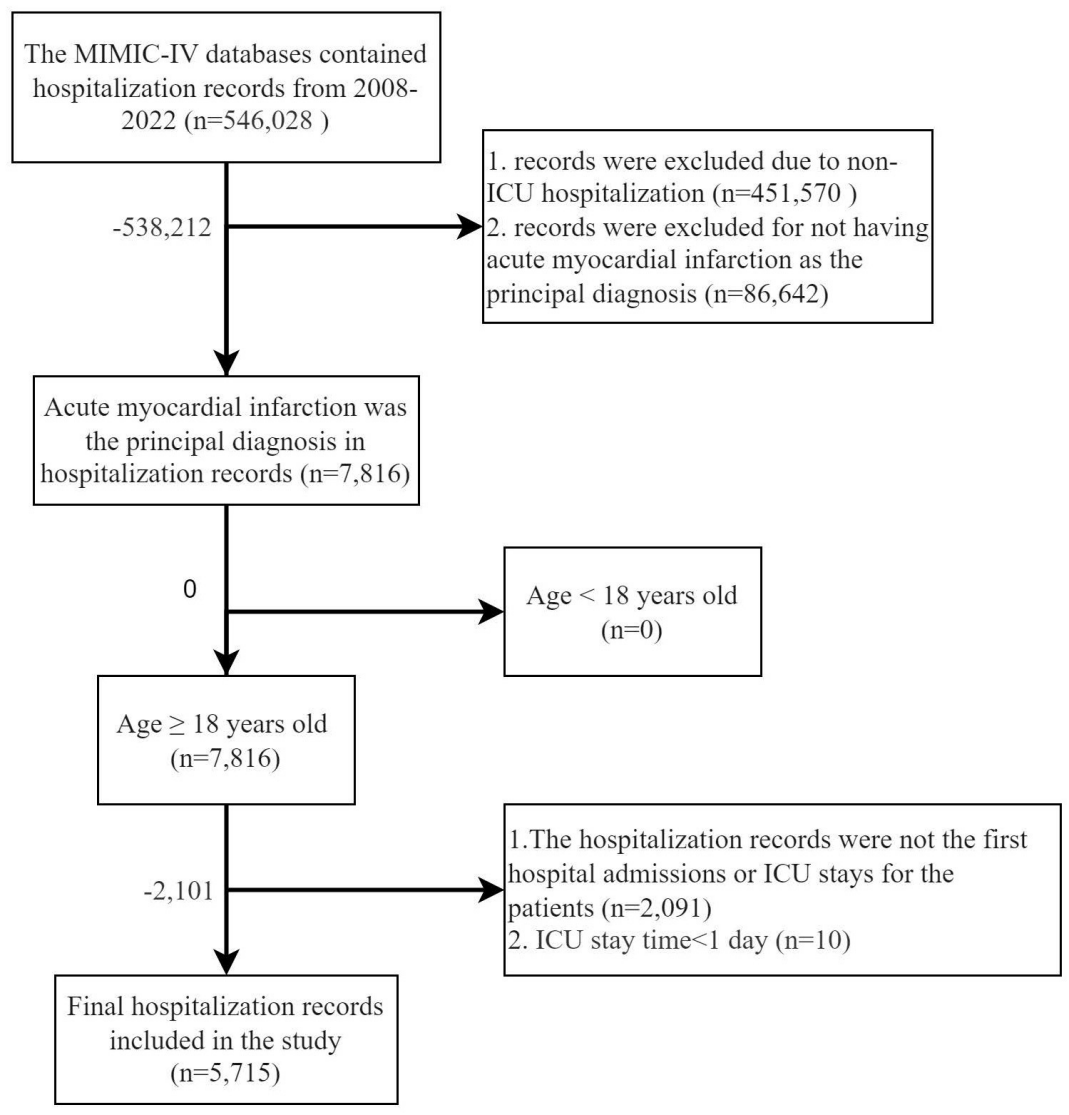
Flow chart of inclusion and exclusion criteria for the target study population of patients with acute myocardial infarction who developed acute kidney injury.

A total of 5,715 patients were included in this study, as illustrated in Figure 1. In the first phase, 1,409 patients were categorized into the Non-AKI group and 1,175 into the Mild-AKI group. After PSM, both groups comprised 656 patients, as detailed in Table 1 and Supplementary Tables S1–S4; In the second phase, 2,584 patients were classified into the Normal-or-mild-AKI group, while 3,131 were assigned to the Moderate-to-severe-AKI group. Following PSM, both groups contained 1,507 patients, as shown in Table 2. In total, 4,306 patients, accounting for 75.36% of the cohort, developed AKI.

**Table 1:** Baseline characteristics before and after propensity score matching of the Non-AKI and Mild-AKI cohorts.

**Covariates**	**Before matching**			**After matching**		
	**Non-AKI (*****N*** = **1,409)**	**Mild-AKI (*****N*** = **1,175)**	**SMD**	**Non-AKI (*****N*** = **617)**	**Mild-AKI (*****N*** = **617)**	**SMD**
Age (years)	**68.00 [59.00, 79.00]**	**71.00 [62.00, 79.00]**	**0.162**	70.00 [61.00, 78.00]	69.00 [61.00, 78.00]	0.038
Gender (Female)	**513 (36.41%)**	**376 (32.00%)**	**0.093**	221 (35.82%)	221 (35.82%)	< 0.001
**ICU score**						
SOFA score	**2.00 [1.00, 4.00]**	**4.00 [2.00, 6.00]**	**0.473**	**4.00 [2.00, 6.00]**	**3.00 [1.00, 5.00]**	**0.277**
**AKI Kidigo**						
0	1,409 (100.00%)	0 (0.00%)	< 0.001	617 (100.00%)	0 (0.00%)	< 0.001
1	0 (0.00%)	1,175 (100.00%)		0 (0.00%)	617 (100.00%)	
2	0 (0.00%)	0 (0.00%)		0 (0.00%)	0 (0.00%)	
3	0 (0.00%)	0 (0.00%)		0 (0.00%)	0 (0.00%)	
**Surgeries and procedures**						
CABG	**186 (13.20%)**	**264 (22.47%)**	**0.244**	129 (20.91%)	118 (19.12%)	0.045
PCI	**193 (13.70%)**	**88 (7.49%)**	**0.203**	51 (8.27%)	57 (9.24%)	0.034
CRRT	**6 (0.43%)**	**19 (1.62%)**	**0.119**	4 (0.65%)	2 (0.32%)	0.047
IABP	**63 (4.47%)**	**97 (8.26%)**	**0.155**	36 (5.83%)	28 (4.54%)	0.058
**Drug use**						
ACEI/ARB	462 (32.79%)	366 (31.15%)	0.035	175 (28.36%)	194 (31.44%)	0.067
Anticoagulant	**1,125 (79.84%)**	**978 (83.23%)**	**0.087**	495 (80.23%)	498 (80.71%)	0.012
Antiplatelet	**1,223 (86.80%)**	**1,055 (89.79%)**	**0.093**	546 (88.49%)	550 (89.14%)	0.021
β-blocker	**1,020 (72.39%)**	**943 (80.26%)**	**0.186**	487 (78.93%)	476 (77.15%)	0.043
Loop diuretic	**542 (38.47%)**	**769 (65.45%)**	**0.561**	**369 (59.81%)**	**302 (48.95%)**	**0.219**
Positive inotropic	**263 (18.67%)**	**159 (13.53%)**	**0.14**	78 (12.64%)	100 (16.21%)	0.102
Spironolactone	21 (1.49%)	27 (2.30%)	0.059	11 (1.78%)	13 (2.11%)	0.023
Statin	**1,141 (80.98%)**	**1,005 (85.53%)**	**0.122**	520 (84.28%)	512 (82.98%)	0.035
Vasopressor	**503 (35.70%)**	**664 (56.51%)**	**0.427**	**329 (53.32%)**	**269 (43.60%)**	**0.196**
**Comorbidities**						
HF	**464 (32.93%)**	**546 (46.47%)**	**0.279**	246 (39.87%)	231 (37.44%)	0.05
AFIB	**112 (7.95%)**	**124 (10.55%)**	**0.09**	59 (9.56%)	48 (7.78%)	0.063
Diabetes	**442 (31.37%)**	**481 (40.94%)**	**0.2**	232 (37.60%)	218 (35.33%)	0.047
Renal disease	**256 (18.17%)**	**356 (30.30%)**	**0.286**	**153 (24.80%)**	**122 (19.77%)**	**0.121**
Liver disease	12 (0.85%)	12 (1.02%)	0.018	8 (1.30%)	6 (0.97%)	0.031
COPD	**154 (10.93%)**	**165 (14.04%)**	**0.094**	77 (12.48%)	80 (12.97%)	0.015
Stroke	**77 (5.46%)**	**88 (7.49%)**	**0.082**	35 (5.67%)	41 (6.65%)	0.04
Malignancy	124 (8.80%)	124 (10.55%)	0.059	56 (9.08%)	59 (9.56%)	0.017
**Vital signs (1st 24 h)**						
MAP (mmHg)	**84.00 [74.00, 96.00]**	**82.00 [72.00, 92.00]**	**0.13**	82.00 [72.00, 93.00]	83.00 [73.00, 93.00]	0.025
Heart rate (bpm)	81.00 [71.00, 93.00]	81.00 [73.00, 92.00]	0.048	81.00 [73.00, 91.00]	80.00 [73.00, 91.00]	0.062
Temperature (°C)	**36.61 [36.39, 36.89]**	**36.56 [36.33, 36.89]**	**0.046**	36.56 [36.33, 36.83]	36.61 [36.39, 36.89]	0.051
**Laboratory tests (1st 24 h)**						
WBC (10^9^/L)	**10.80 [8.20, 13.60]**	**11.00 [8.30, 14.90]**	**0.057**	11.00 [8.40, 14.30]	10.80 [8.20, 14.00]	0.064
Hemoglobin (g/dl)	**11.60 [9.80, 13.30]**	**10.70 [9.00, 12.60]**	**0.283**	**10.90 [9.40, 12.70]**	**11.40 [9.60, 13.10]**	**0.157**
Platelet (10^9^/L)	**202.00 [156.00, 250.00]**	**187.00 [138.00, 240.50]**	**0.117**	189.00 [136.00, 242.00]	192.00 [149.00, 249.00]	0.093
Sodium (mmol/L)	**138.00 [135.00, 140.00]**	**137.00 [134.00, 140.00]**	**0.133**	**137.00 [135.00, 139.00]**	**138.00 [135.00, 140.00]**	**0.118**
Potassium (mmol/L)	**4.20 [3.90, 4.60]**	**4.30 [3.90, 4.80]**	**0.189**	**4.30 [3.90, 4.80]**	**4.20 [3.90, 4.60]**	**0.125**
Bicarbonate (mmol/L)	**23.00 [21.00, 25.00]**	**23.00 [20.00, 25.00]**	**0.106**	23.00 [21.00, 25.00]	23.00 [21.00, 25.00]	0.074
Chloride (mmol/L)	104.00 [101.00, 107.00]	104.00 [101.00, 107.00]	0.095	104.00 [101.00, 107.00]	104.00 [101.00, 107.00]	0.027
BUN (mg/dl)	**17.00 [13.00, 26.00]**	**20.00 [15.00, 33.00]**	**0.196**	**18.00 [14.00, 31.00]**	**17.00 [13.00, 25.00]**	**0.184**
Creatinine (mg/dl)	**0.90 [0.80, 1.20]**	**1.00 [0.80, 1.60]**	**0.282**	**1.00 [0.80, 1.40]**	**0.90 [0.80, 1.20]**	**0.204**
eGFR (ml/min/1.73m^2^)	**77.02 [55.06, 97.32]**	**65.80 [39.71, 88.27]**	**0.334**	**70.12 [44.62, 91.88]**	**75.23 [54.05, 97.32]**	**0.216**
BNP (tested)	45 (3.19%)	45 (3.83%)	0.035	26 (4.21%)	22 (3.57%)	0.034
TNT (tested)	**946 (67.14%)**	**648 (55.15%)**	**0.248**	354 (57.37%)	376 (60.94%)	0.073
CK-MB (tested)	**652 (46.27%)**	**449 (38.21%)**	**0.164**	240 (38.90%)	261 (42.30%)	0.069

CABG, coronary artery bypass grafting; PCI, percutaneous coronary intervention; CRRT, continuous renal replacement therapy; IABP, intra-aortic balloon pump; ACEI/ARB, angiotensin-converting enzyme inhibitor/angiotensin II receptor blocker; HF, heart failure; AFIB, atrial fibrillation; COPD, chronic obstructive pulmonary disease; MAP, mean arterial pressure; WBC, white blood cell; BUN, blood urea nitrogen; TNT, troponin T; CK-MB, creatine kinase-muscle/brain; eGFR, estimated glomerular filtration rate; CK-MB, creatine kinase-MB. Values are presented as mean (standard deviation) or median [Q1, Q3] for continuous variables and number (percentage) for categorical variables. Variables in bold have *p*-value < 0.05.

**Table 2:** Baseline characteristics before and after propensity score matching of the Normal-or-mild-AKI and Moderate-to-severe-AKI cohorts.

**Covariates**	**Before matching**			**After matching**		
	**Normal-or-mild-AKI (*****N*** = **2,584)**	**Moderate-to-severe-AKI (*****N*** = **3,131)**	**SMD**	**Normal-or-mild-AKI (*****N*** = **1,449)**	**Moderate-to-severe-AKI (*****N*** = **1,449)**	**SMD**
Age (years)	**69.00 [60.00, 79.00]**	**73.00 [64.00, 81.00]**	**0.218**	72.00 [63.00, 80.00]	71.00 [63.00, 80.00]	0.026
Gender (Female)	889 (34.40%)	1,105 (35.29%)	0.019	527 (36.37%)	514 (35.47%)	0.019
**ICU score**						
SOFA score	**3.00 [1.00, 5.00]**	**5.00 [3.00, 8.00]**	**0.538**	**4.00 [2.00, 6.00]**	**4.00 [2.00, 6.00]**	**0.214**
**AKI KIDIGO**						
0	**1,409 (54.53%)**	**0 (0.00%)**	**3.88**	**656 (45.27%)**	**0 (0.00%)**	**3.65**
1	**1,175 (45.47%)**	**0 (0.00%)**		**793 (54.73%)**	**0 (0.00%)**	
2	**0 (0.00%)**	**2065 (65.95%)**		**0 (0.00%)**	**1,141 (78.74%)**	
3	**0 (0.00%)**	**1066 (34.05%)**		**0 (0.00%)**	**308 (21.26%)**	
**Surgeries and procedures**						
CABG	450 (17.41%)	530 (16.93%)	0.013	288 (19.88%)	310 (21.39%)	0.038
PCI	**281 (10.87%)**	**217 (6.93%)**	**0.139**	106 (7.32%)	110 (7.59%)	0.011
CRRT	**25 (0.97%)**	**130 (4.15%)**	**0.203**	22 (1.52%)	16 (1.10%)	0.036
IABP	**160 (6.19%)**	**442 (14.12%)**	**0.265**	140 (9.66%)	111 (7.66%)	0.071
**Drug use**						
ACEI/ARB	828 (32.04%)	1054 (33.66%)	0.034	478 (32.99%)	471 (32.51%)	0.01
Anticoagulant	**2,103 (81.39%)**	**2,821 (90.10%)**	**0.251**	1,241 (85.65%)	1,202 (82.95%)	0.074
Antiplatelet	**2,278 (88.16%)**	**2,845 (90.87%)**	**0.088**	1,293 (89.23%)	1,283 (88.54%)	0.022
β-Blocker	**1,963 (75.97%)**	**2,489 (79.50%)**	**0.085**	1,159 (79.99%)	1,167 (80.54%)	0.014
Loop diuretic	**1,311 (50.74%)**	**2,251 (71.89%)**	**0.445**	**965 (66.60%)**	**849 (58.59%)**	**0.166**
Positive inotropic	422 (16.33%)	554 (17.69%)	0.036	202 (13.94%)	204 (14.08%)	0.004
Spironolactone	48 (1.86%)	78 (2.49%)	0.043	34 (2.35%)	32 (2.21%)	0.009
Statin	**2,146 (83.05%)**	**2,743 (87.61%)**	**0.129**	1,254 (86.54%)	1,246 (85.99%)	0.016
Vasopressor	**1,167 (45.16%)**	**2,146 (68.54%)**	**0.486**	**850 (58.66%)**	**719 (49.62%)**	**0.182**
**Comorbidities**						
HF	**1,010 (39.09%)**	**1,739 (55.54%)**	**0.334**	**696 (48.03%)**	**621 (42.86%)**	**0.104**
AFIB	**236 (9.13%)**	**492 (15.71%)**	**0.201**	188 (12.97%)	179 (12.35%)	0.019
Diabetes	**923 (35.72%)**	**1,303 (41.62%)**	**0.121**	580 (40.03%)	575 (39.68%)	0.007
Renal disease	**612 (23.68%)**	**1,003 (32.03%)**	**0.187**	**429 (29.61%)**	**367 (25.33%)**	**0.096**
Liver disease	**24 (0.93%)**	**66 (2.11%)**	**0.097**	16 (1.10%)	16 (1.10%)	< 0.001
COPD	**319 (12.35%)**	**489 (15.62%)**	**0.094**	206 (14.22%)	186 (12.84%)	0.04
Stroke	**165 (6.39%)**	**373 (11.91%)**	**0.193**	131 (9.04%)	124 (8.56%)	0.017
Malignancy	248 (9.60%)	345 (11.02%)	0.047	144 (9.94%)	148 (10.21%)	0.009
**Vital signs (1st 24 h)**						
MAP (mmHg)	**83.00 [73.00, 94.00]**	**81.00 [71.00, 94.00]**	**0.045**	82.00 [72.00, 93.00]	82.00 [72.00, 93.00]	0.029
Heart rate (bpm)	**81.00 [72.00, 92.00]**	**84.00 [74.00, 97.00]**	**0.148**	82.00 [73.00, 94.00]	81.00 [73.00, 93.00]	0.052
Temperature (°C)	**36.61 [36.39, 36.89]**	**36.61 [36.33, 36.94]**	**0.037**	36.56 [36.33, 36.89]	36.61 [36.33, 36.89]	0.009
**Laboratory tests (1st 24 h)**						
WBC (10^9^/L)	**10.90 [8.30, 14.12]**	**12.20 [9.10, 16.30]**	**0.152**	**11.70 [8.80, 15.30]**	**11.20 [8.60, 14.70]**	**0.102**
Hemoglobin (g/dl)	**11.20 [9.40, 13.00]**	**10.90 [9.10, 12.70]**	**0.098**	10.80 [9.20, 12.60]	10.90 [9.30, 12.60]	0.032
Platelet (10^9^/L)	195.00 [146.00, 246.25]	192.00 [145.00, 251.00]	0.016	189.00 [142.00, 245.00]	188.00 [144.00, 242.00]	0.038
Sodium (mmol/L)	137.00 [135.00, 140.00]	137.00 [134.00, 140.00]	0.026	137.00 [134.00, 140.00]	137.00 [135.00, 140.00]	0.01
Potassium (mmol/L)	4.20 [3.90, 4.70]	4.30 [3.90, 4.80]	0.033	4.30 [3.90, 4.70]	4.30 [3.90, 4.70]	0.037
Bicarbonate (mmol/L)	**23.00 [21.00, 25.00]**	**22.00 [20.00, 25.00]**	**0.128**	**23.00 [20.00, 25.00]**	**23.00 [21.00, 25.00]**	**0.084**
Chloride (mmol/L)	**104.00 [101.00, 107.00]**	**104.00 [100.00, 107.00]**	**0.106**	104.00 [100.00, 107.00]	104.00 [101.00, 107.00]	0.032
BUN (mg/dl)	**18.00 [14.00, 29.00]**	**23.00 [16.00, 36.00]**	**0.217**	**21.00 [15.00, 33.00]**	**19.00 [14.00, 30.00]**	**0.091**
Creatinine (mg/dl)	**1.00 [0.80, 1.40]**	**1.10 [0.90, 1.70]**	**0.215**	**1.10 [0.80, 1.60]**	**1.00 [0.80, 1.40]**	**0.059**
eGFR (ml/min/1.73m^2^)	**72.08 [46.56, 95.29]**	**57.75 [35.26, 81.81]**	**0.312**	**62.86 [39.31, 86.25]**	**67.85 [45.23, 92.68]**	**0.123**
BNP (tested)	**90 (3.48%)**	**170 (5.43%)**	**0.094**	63 (4.35%)	60 (4.14%)	0.01
TNT (tested)	**1,594 (61.69%)**	**2,093 (66.85%)**	**0.108**	841 (58.04%)	859 (59.28%)	0.025
CK-MB (tested)	**1,101 (42.61%)**	**1,527 (48.77%)**	**0.124**	610 (42.10%)	608 (41.96%)	0.003

CABG, coronary artery bypass grafting; PCI, percutaneous coronary intervention; CRRT, continuous renal replacement therapy; IABP, intra-aortic balloon pump; ACEI/ARB: angiotensin-converting enzyme inhibitor/angiotensin II receptor blocker; HF, heart failure; AFIB, atrial fibrillation; COPD, chronic obstructive pulmonary disease; MAP, mean arterial pressure; WBC, white blood cell; BUN, blood urea nitrogen; TNT, troponin T; CK-MB, creatine kinase-muscle/brain; eGFR, estimated glomerular filtration rate; CK-MB, creatine kinase-MB. Values are presented as mean (standard deviation) or median [Q1, Q3] for continuous variables and number (percentage) for categorical variables. Variables in bold have *p*-value < 0.05.

### Doubly robust analysis

**Figure 2:**
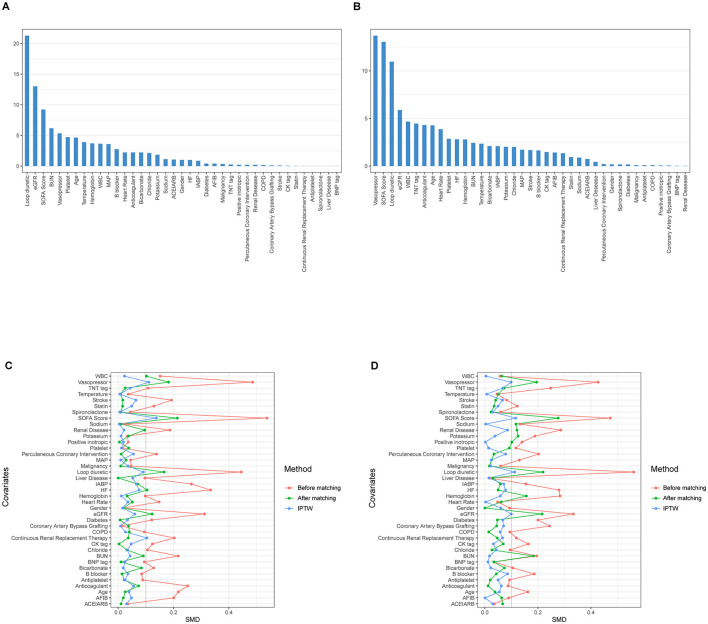
The relative influence factors evaluate the discriminative power of the 39 covariates within the propensity score model in predicting acute kidney injury (AKI) outcomes (the higher the value in the bar graph, the more important the variable). **(A)** For Non-AKI and Mild-AKI; **(B)** For Normal-or-mild-AKI and Moderate-to-severe-AKI; Change in standardized mean difference (SMD) of cohorts before and after propensity score matching: the red curve represents pre-matching, the green curve represents post-matching, and the blue curve represents inverse probability of treatment weighting (IPTW) adjustment. The smaller the curve fluctuation, the better the data quality. **(C)** For Non-AKI and Mild-AKI; **(D)** For Normal-or-mild-AKI and Moderate-to-severe-AKI.

A propensity score model was initially developed using 39 covariates through GBM. Figure 2 illustrates the relative contributions of each covariate to the calculated propensity scores. Figure 2A highlights that the most significant covariates distinguishing the Non-AKI and Mild-AKI groups include the use of loop diuretics, eGFR, SOFA score, BUN, and vasopressor use, all closely associated with the onset of AKI; Figure 2B shows that the key covariates differentiating the Normal-or-mild-AKI group from the Moderate-to-severe-AKI group are vasopressor use, SOFA score, loop diuretics, eGFR, and WBC, all of which are strongly linked to the progression to Moderate-to-severe AKI.

Using the estimated propensity scores, IPTW was applied to standardize differences between the Non-AKI and Mild-AKI groups, as well as between the Normal-or-mild-AKI and Moderate-to-severe-AKI groups. Details are presented in Table 1 and Figures 2C, D. In the first analysis, most covariates in the weighted cohorts were comparable or balanced between the Non-AKI and Mild-AKI groups, with some exceptions: SOFA score, loop diuretics, vasopressor use, renal disease, hemoglobin, sodium, potassium, BUN, creatinine, and eGFR; In the second analysis, most covariates were similarly balanced between the Normal-or-mild-AKI and Moderate-to-severe-AKI groups, with exceptions for SOFA score, loop diuretics, vasopressor use, heart failure, renal disease, WBC, bicarbonate, BUN, creatinine, and eGFR.

To address the residual imbalance in covariates within the weighted cohorts, several regression models were constructed using doubly robust estimation.

### Outcomes and sensitivity studies

**Figure 3:**
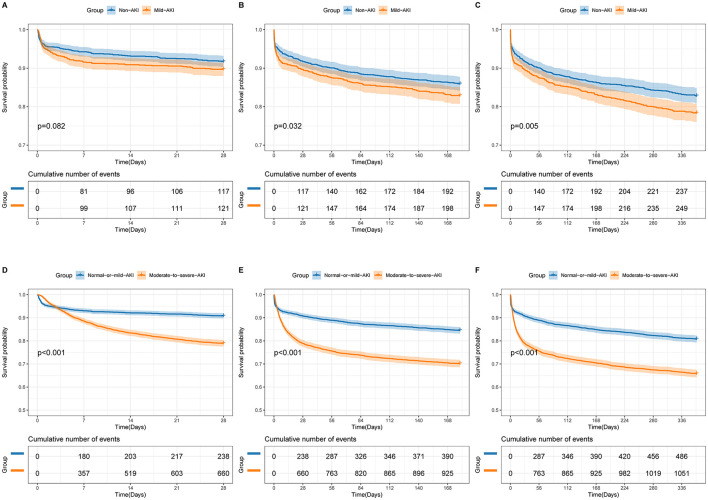
Unadjusted Kaplan-Meier survival curves for 28-day mortality **(A, D)**, 180-day mortality **(B, E)**, and 1-year mortality **(C, F)**. **(A–C)** represent comparisons between Non-AKI and Mild-AKI groups, while **(D–F)** represent comparisons between Normal-or-mild-AKI and Moderate-to-severe-AKI groups. The shaded areas in the graphs indicate the 95% confidence interval.

The doubly robust analysis revealed no significant differences in short- or long-term outcomes between patients with Non-AKI and Mild-AKI (28-day mortality: OR 0.97, 95% CI 0.68–1.38, *p* = 0.854; 180-day mortality: HR 0.94, 95% CI 0.76–1.18, *p* = 0.618; 1-year mortality: HR 0.98, 95% CI 0.81–1.20, *p* = 0.857); However, when comparing the Normal-or-mild-AKI group with the Moderate-to-severe-AKI group, patients with Moderate-to-severe-AKI had a significantly worse prognosis (28-day mortality: OR 1.67, 95% CI 1.36–2.05, *p* < 0.001; 180-day mortality: HR 1.06, 95% CI 1.02–1.10, *p* < 0.001; 1-year mortality: HR 1.22, 95% CI 1.07–1.38, *p* < 0.001). As shown in Table 3, Supplementary Tables S5–S28 and Figure 3, sensitivity analyses consistently confirmed these findings across all estimation models.

**Table 3:** Primary outcome with different models for two parts.

**Methods**	**Non-AKI vs. Mild-AKI**		**Normal-or-mild-AKI vs. Moderate-to-severe-AKI**	
	**Result**	***p*** **-value**	**Result**	***p*** **-value**
**28-day mortality**				
Log-rank test [HR (95% CI)]	1.25 (0.97, 1.62)	0.082	**2.38 (2.09, 2.71)**	< 0.001
Multivariate Logistic model adjusted with all covariates [OR (95% CI)]	0.94 (0.64, 1.36)	0.736	**2.01 (1.63, 2.48)**	< 0.001
Multivariate Logistic model adjusted with unbalanced covariates [OR (95% CI)]	0.9 (0.69, 1.17)	0.444	**1.87 (1.62, 2.15)**	< 0.001
Survey-weighted GLM model adjusted with all covariates using IPTW [OR (95% CI)]	0.9 (0.62, 1.31)	0.588	**1.87 (1.49, 2.33)**	< 0.001
Survey-weighted GLM model adjusted with unbalanced covariates using IPTW [OR (95% CI)]	0.97 (0.68, 1.38)	0.854	**1.67 (1.36, 2.05)**	< 0.001
**180-day mortality**				
Log-rank test [HR (95% CI)]	**1.24 (1.02, 1.51)**	< 0.05	**2.10 (1.88, 2.34)**	< 0.001
Multivariate COX model adjusted with all covariates [HR (95% CI)]	0.95 (0.77, 1.18)	0.671	**1.09 (1.06, 1.13)**	< 0.001
Multivariate Cox model adjusted with unbalanced covariates [HR (95% CI)]	1.02 (0.82, 1.27)	0.839	**1.07 (1.03, 1.10)**	< 0.001
Survey-weighted Cox model adjusted with all covariates using IPTW [HR (95% CI)]	0.89 (0.71, 1.11)	0.309	**1.07 (1.03, 1.11)**	< 0.001
Survey-weighted Cox model adjusted with unbalanced covariates using IPTW [HR (95% CI)]	0.94 (0.76, 1.18)	0.618	**1.06 (1.02, 1.10)**	< 0.01
**1-year mortality**				
Log-rank test [HR (95% CI)]	**1.29 (1.08, 1.54)**	< 0.01	**1.97 (1.78, 2.17)**	< 0.001
Multivariate Cox model adjusted with all covariates [HR (95% CI)]	0.99 (0.81, 1.19)	0.879	**1.37 (1.22, 1.54)**	< 0.001
Multivariate Cox model adjusted with unbalanced covariates [HR (95% CI)]	1.05 (0.87, 1.27)	0.626	**1.28 (1.14, 1.44)**	< 0.001
Survey-weighted Cox model adjusted with all covariates using IPTW [HR (95% CI)]	0.93 (0.76, 1.14)	0.491	**1.26 (1.11, 1.43)**	< 0.001
Survey-weighted Cox model adjusted with unbalanced covariates using IPTW [HR (95% CI)]	0.98 (0.81, 1.20)	0.857	**1.22 (1.07, 1.38)**	< 0.01

Statistical analyses of different models with *p*-value < 0.05 were displayed in bold. HR, odds ratio; HR, hazard ratio; CI, confidence interval; IPTW, inverse probability of treatment weighting.

### Subgroup analysis

**Figure 4:**
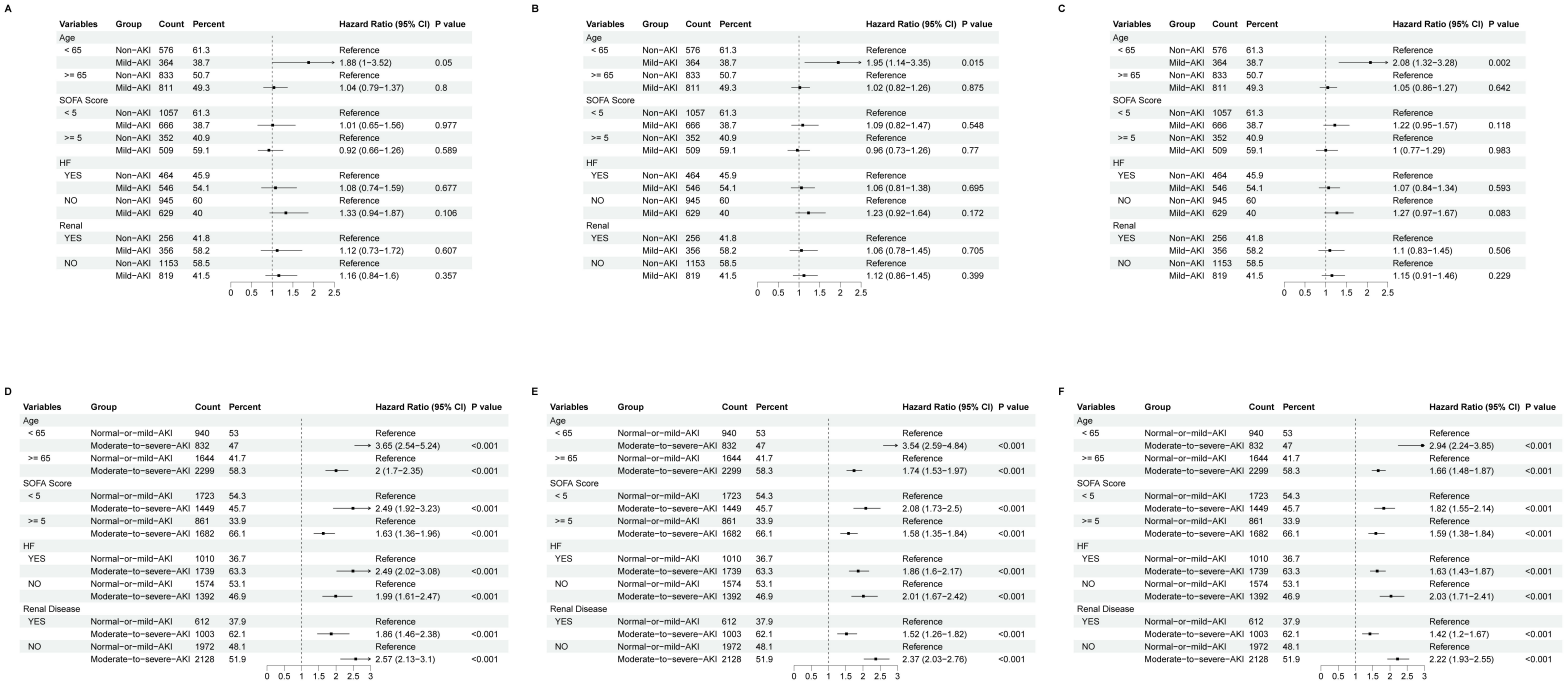
Forest plot of subgroup analysis for 28-day mortality **(A, D)**, 180-day mortality **(B, E)**, and 1-year mortality **(C, F)**. **(A–C)** represent comparisons between Non-AKI and Mild-AKI groups, while **(D–F)** represent comparisons between Normal-or-mild-AKI and Moderate-to-severe-AKI groups. The dashed lines in the plots indicate the null effect line, and any result intersecting the null effect line suggests no significant difference.

We performed a subgroup analysis stratified by age (< 65 or ≥65 years), SOFA score (< 5 or ≥5), heart failure status, and renal disease status. Within the Non-AKI and Mild-AKI group, patients under 65 years with Mild-AKI exhibited a significantly higher risk of 180-day and 1-year mortality compared to those without AKI. However, no significant differences in outcomes were observed between Non-AKI and Mild-AKI patients within the other subgroups; In the comparison between the Normal-or-mild-AKI and Moderate-to-severe-AKI groups, patients with Moderate-to-severe-AKI consistently showed significantly worse outcomes compared to those with Normal-or-mild-AKI across all subgroups. These findings are illustrated in Figure 4.

## Discussion

AMI is a leading cause of global mortality and morbidity. Patients with AMI are highly susceptible to complications such as AKI, which is common in critically ill patients and linked to worse clinical outcomes, including higher morbidity and mortality (22). While the association between AKI and adverse outcomes is well-documented, the impact of varying severities of AKI on the prognosis of AMI patients remains not fully understood. Addressing this crucial knowledge gap can inform targeted interventions and improve clinical decision-making. Our study uses advanced statistical methods, such as doubly robust estimation and rigorous sensitivity analyses, to explore the short- and long-term outcomes of ICU patients with different severities of AKI following AMI. This comprehensive approach aims to enhance clinical practices and guide future research by providing a detailed understanding of AKI severity's prognostic implications.

Cardiorenal syndrome (CRS) is a complex disorder characterized by bidirectional interactions between cardiac and renal dysfunction, mediated by multiple molecular mechanisms. It is a major cause of AKI in patients with AMI (20). Oxidative stress and inflammation represent central pathways in CRS pathogenesis. Activation of the NF-κB signaling pathway promotes the production of pro-inflammatory cytokines (e.g., IL-6, TNF-α) and oxidative stress markers (e.g., NOX2, iNOS), contributing to tissue damage in both organs (23, 24). Concurrently, impairment of the Nrf2 antioxidant pathway reduces the expression of cytoprotective enzymes (e.g., HO-1, MnSOD), exacerbating oxidative injury (20). These processes are evident across all CRS subtypes, as demonstrated in animal models of myocardial infarction (CRS1) and chronic kidney disease (CRS2) (25, 26). The renin-angiotensin-aldosterone system (RAAS) is hyperactivated in CRS, leading to vasoconstriction, sodium retention, and fibrosis. Upregulation of ACE and AT1R, coupled with downregulation of protective AT2R and MasR, has been observed in experimental models (27). RAAS inhibitors (e.g., ACEIs/ARBs) reduce urinary podocin loss in CRS2 patients, indicating glomerular protection (28). Moreover, the TGF-β1/Smad pathway mediates fibrosis through collagen deposition and epithelial-mesenchymal transition. Studies in CRS rats show elevated TGF-β1 and phosphorylated Smad3 in cardiac and renal tissues, which are attenuated by empagliflozin and dapagliflozin (29). Aberrant Wnt/β-catenin signaling contributes to cardiac hypertrophy and renal fibrosis. In CRS2 models, activation of β-catenin promotes pro-fibrotic gene expression (e.g., Twist, Snail1), while its inhibition with ICG-001 ameliorates organ damage (30). Gut microbiota dysbiosis further exacerbates CRS by producing uremic toxins (e.g., TMAO), which enhance inflammation and fibrosis via NF-κB and TGF-β1 pathways (31, 32). Noncoding RNAs, such as miR-21 and lncRNA ANRIL, also play roles by modulating fibrosis (e.g., targeting PPARα) and inflammasome activation (33, 34).

Research indicates that the incidence of AKI among ICU patients ranges from 12.1% to 60.93% (1). However, our study found a significantly higher AKI incidence of 75.35% in patients with AMI. We believe this discrepancy may stem from our more sensitive method of detecting AKI. In our study, each laboratory test and every fluctuation in fluid input/output were dynamically monitored throughout the hospitalization period, allowing for earlier detection of AKI. This increased sensitivity, as described in our study design, likely contributed to the higher incidence we observed. Supporting this, a study involving 1,050 AMI patients demonstrated that using the KDIGO criteria identified significantly more cases of AKI compared to the RIFLE criteria, suggesting that KDIGO is more sensitive for detecting AKI in AMI patients (15). Additionally, Kanic et al. (35) found that even minor rises in serum creatinine and progressive increases in AKI severity, as evaluated by the KDIGO criteria, were associated with poorer long-term outcomes in AMI patients. This underscores the importance of employing more sensitive methods, like KDIGO, to detect AKI in this population.

Compared to patients with Mild-AKI, those Non-AKI used loop diuretics and vasopressors more frequently, and they exhibited lower eGFR, higher SOFA scores, and elevated BUN levels. These five variables were identified as the most significant during the PSM process. The use of loop diuretics, lower eGFR, higher BUN levels, and elevated SOFA scores all indicate poorer renal function. Additionally, the frequent use of vasopressors suggests a higher incidence of hypotensive states, which can lead to renal ischemia and further kidney function deterioration (36).

In the PSM process comparing the Normal-or-mild-AKI and Moderate-to-severe-AKI groups, the five most significant variables were vasopressor use, SOFA score, loop diuretics, eGFR, and WBC count. The first four variables were consistent with the findings in the Non-AKI group. However, the WBC count was notably higher in the Moderate-to-severe-AKI group. Elevated leukocyte levels, particularly WBC, play a critical role in the pathophysiology of AKI, involving complex immunopathological interactions. These include mechanisms such as damage-associated molecular patterns (DAMPs), pathogen-associated molecular patterns (PAMPs), oxidative stress, hypoxia-inducible factors, the complement system, and various immune cells like dendritic cells, neutrophils, lymphocytes, and macrophages (37). A study by Chen et al. found that in AMI patients, the percentage of neutrophils in peripheral blood (NEUT%) was positively correlated with both the incidence of AKI and short-term all-cause mortality (38). Elevated serum calcium can induce renal vasoconstriction, reducing renal blood flow and causing tubular injury, while hypocalcemia may indicate the severity of cardiac dysfunction and renal impairment. Previous research (39) has demonstrated that acute kidney injury occurs more frequently in patients with ST-elevation myocardial infarction complicated by cardiogenic shock, leading to poor short-term clinical outcomes. A recent study (40) utilizing the MIMIC database developed a predictive model for AKI risk in AMI patients. Their model identified estimated glomerular filtration rate, creatinine, blood urea nitrogen, cardiogenic shock, and creatine-kinase myocardial band as the five most significant predictors. These findings are largely consistent with our results.

It is important to note that certain variables remained unbalanced between groups despite PSM. To minimize the impact of these variables on study outcomes, we performed corrections in subsequent modeling, though it remains necessary to discuss why statistical methods struggled to eliminate imbalances in these factors. Seven variables showed persistent intergroup imbalance in both analyses (Non-AKI vs. Mild-AKI and Normal/mild-AKI vs. Moderate-severe-AKI): SOFA score, loop diuretic use, vasopressor administration, preexisting renal disease, BUN, creatinine, and eGFR. These metrics are inherently linked to renal function, explaining why baseline disparities for such variables persisted even after PSM—patients with divergent renal profiles inherently exhibit unequal baselines for these parameters. The renal subcomponent of the SOFA score directly assesses renal function, while a history of preexisting renal disease characterizes chronic renal status. Loop diuretic use often reflects fluid overload, a hallmark of renal dysfunction, whereas vasopressor therapy typically indicates hypotension that may compromise renal perfusion. BUN, creatinine, and eGFR serve as direct renal function biomarkers: elevations in BUN, creatinine and declines in eGFR are sensitive indicators of deteriorating renal health.

Additionally, imbalance persisted in several indices across groups, presumably because statistical methods like PSM—being baseline characteristic-matched—cannot eliminate differences in indices strongly associated with the disease itself. In the Non-AKI vs. Mild-AKI group, renal concentrating dysfunction in AKI causes hyponatremia, while tubular potassium excretion impairment leads to hyperkalemia, contributing to data imbalance (41). Anemia (low hemoglobin) acts as both a risk factor for AKI (e.g., reduced oxygen-carrying capacity exacerbates renal injury during ischemia) and a consequence (e.g., decreased renal erythropoietin secretion), forming a bidirectional relationship (42).

In the Normal/mild-AKI vs. Moderate-severe-AKI group, heart failure impairs cardiac pumping, reducing renal perfusion, triggering renal vasoconstriction, and activating the renin-angiotensin-aldosterone system (RAAS) to induce/worsen AKI (43), making these patients more prone to moderate-severe AKI. WBC counts increase with infection, inflammation, and stress, and higher AKI severity correlates with greater infection probability and stress response (37), leading to uneven WBC distribution. As a key acid-base buffer regulated by the kidneys, bicarbonate metabolism is disrupted in AKI: varying degrees of renal injury across AKI severities cause differential impairment in bicarbonate reabsorption/secretion, and bicarbonate levels inversely correlate with AKI incidence and prognosis (44).

Numerous studies have examined the impact of AKI on prognosis in patients with AMI. Skalsky et al. (45) found that AMI patients with stage 1 AKI who did not recover within 48 h, as well as those with stage 2–3 AKI without recovery within 96 h, had a significantly higher risk of mortality. However, their diagnosis and staging of AKI were based solely on serum creatinine levels, without considering the diagnostic significance of urine output. Similarly, Kanic et al. (35) reported that the incidence of AKI among AMI patients undergoing PCI was 8.5%. During an average follow-up of 4.2 ± 3.0 years, the mortality rates were 50.3% for stage 1 AKI, 56.9% for stage 2, and 87.2% for stage 3. The hazard ratios for all-cause mortality were 1.77, 1.85, and 6.30 for stages 1, 2, and 3, respectively, compared to patients without AKI. In another study, Sun et al. observed 1,371 AMI patients and found that the severity of AKI, as classified by the KDIGO criteria, was an independent risk factor for 30-day mortality. Stage 3 AKI was also identified as an independent predictor of mortality between 30 days and 5 years. However, like previous studies, their definition of AKI relied solely on serum creatinine levels, without incorporating assessments of GFR or urine output. A review by Kaltsas et al. (11) summarized key studies on AKI complicating AMI, emphasizing that all studies consistently showed AKI worsened patient outcomes, increasing mortality by two- to threefold both within the first 30 days and throughout the first year after the acute event. Furthermore, a study (46) identified serum calcium levels as a strong predictor of AKI in AMI patients. Consequently, we set our outcome measures at 28 days, 180 days, and 1 year to better evaluate the long-term prognostic characteristics of this patient population.

We utilized a more sensitive dynamic assessment method based on the KDIGO criteria to evaluate the occurrence of AKI in patients with AMI. As shown in Table 3, no significant differences in prognosis were observed between the Non-AKI and Mild-AKI groups in the multivariable-adjusted models. However, the Log-rank test indicated differences in 180-day and 1-year mortality rates, suggesting that these differences may have been driven by other covariates rather than AKI itself. Further subgroup analysis identified age as a potential contributing factor. Specifically, patients under 65 years with Mild-AKI had worse outcomes compared to those without AKI. As reported in a study (47), younger patients in the ICU are more sensitive to nephrotoxic drugs (e.g., vancomycin and calcineurin inhibitors), which significantly deteriorate the prognosis of young AKI patients. Our analysis also suggests that physicians might adopt more conservative treatment strategies for these patients, assuming that younger individuals have stronger renal compensatory capacity. This approach may lead to progression of mild AKI or delayed control of systemic effects, thereby impacting patient outcomes. Across all models for the three outcome events, patients with Moderate-to-severe AKI consistently had worse outcomes compared to those with Normal-or-mild AKI. Sensitivity analyses confirmed the robustness of these findings.

Patients with Mild-AKI, referred to as “subclinical AKI,” only reached stage 1 AKI during hospitalization and did not experience adverse prognostic effects. In contrast, many patients initially classified as stage 1 AKI progressed to stages 2–3, leading to significantly worse outcomes compared to those who remained at AKI stages 0–1. The dynamic evaluation of AKI stages is clinically significant, as it allows for early detection of “subclinical AKI,” enabling timely intervention to prevent “conversion” to Moderate-to-severe AKI. The risk factors for progression may include the use of vasopressors, loop diuretics, higher SOFA scores, lower eGFR, and elevated WBC counts.

The treatment of CRS remains challenging. Diuretics, a mainstay in managing fluid overload, have uncertain long-term benefits. High-dose intermittent furosemide seems safe and effective in acute heart failure, but its impact on severe kidney disease is unclear. Ultrafiltration shows promise in some aspects like weight loss, yet its overall efficacy is still debated. CARRESS-HF indicated that ultrafiltration might not be the best primary treatment for type 1 CRS. While neurohormonal modulation therapies such as vasopressin antagonists and nesiritide have not significantly improved clinical outcomes in large-scale trials (48), sacubitril/valsartan has demonstrated renal protective effects in patients with cardiorenal syndrome (49). Furthermore, both traditional vasopressin antagonists and sacubitril/valsartan have been proven to be safe in clinical use. RAAS inhibitors are beneficial for some patients with CRS, yet they carry risks like hyperkalemia. β-adrenergic blockers have shown efficacy in reducing mortality in heart failure, but their use in CRS patients needs more evidence (48). Meanwhile, research has shown (50) that psychological interventions for patients with AKI can help improve their clinical outcomes.

Future research should focus on identifying additional influential factors and developing machine learning and deep learning models to predict the risk of moderate-to-severe AKI in AMI patients, and develop targeted effective treatment strategies to improve patient outcomes.

## Limitation

While MIMIC-IV's data provide detailed records of clinical information for critically ill patients, the single-center and retrospective design warrant careful consideration of generalizability. Clinical data from this center may differ from those in community, rural, or international settings, potentially influencing outcome estimates—particularly for subgroups underrepresented in the dataset.

The retrospective design introduces risks of selection bias and unmeasured confounding factors. Although rigorous statistical methods were used to mitigate these limitations, residual confounding from unrecorded variables (e.g., family medical history, socioeconomic status) cannot be fully eliminated, which may affect the robustness of our findings.

External validation in independent cohorts remains essential to confirm the stability of these results. Future research should prioritize prospective multicenter studies across diverse healthcare systems to evaluate consistency across populations with differing baseline risks and care environments. Such efforts will enhance the generalizability of this study's findings and help provide a reliable theoretical foundation for clinical practice, ensuring these insights can inform real-world medical decision-making.

## Conclusion

Patients with Mild-AKI can be more accurately described as having “subclinical AKI,” as their prognosis is often comparable to that of Non-AKI patients. However, the prognosis for those with Moderate-to-severe AKI is significantly worse than for patients with Normal-or-mild AKI. This indicates that if “subclinical AKI” undergoes a “conversion” to Moderate-to-severe AKI during hospitalization, the patient's prognosis will deteriorate considerably. Therefore, the dynamic and sensitive early identification of “subclinical AKI” and its potential “conversion” to Moderate-to-severe AKI is of great importance for timely intervention and improved outcomes.

## Data Availability

The datasets presented in this article are not readily available due to MIMIC dataset requiring principal investigator approval for access, the data cannot be made publicly available. Requests to access the datasets should be directed to https://www.physionet.org/content/mimiciv/3.0/.
