# Supplementary material for: Acute kidney injury after myocardial infarction: prognostic implications via dual robust methods
Source: Front Med (Lausanne). 2025 Jul 22;12:1555478. doi: 10.3389/fmed.2025.1555478 (PMC12321540; doi:10.3389/fmed.2025.1555478)
Supplement: Supplementary file 1 [file Table_1.docx]

Supplementary Table 1. Standardized mean difference (SMD) of covariates before and after propensity score matching of the Non-AKI and Mild-AKI cohorts

| Characteristic | Before matcing | After matcing |
| --- | --- | --- |
| SMD ≤ 0.1 | 15 | 29 |
| SMD > 0.1 | 24 | 10 |
| Total number of covariates | 39 | 39 |

Supplementary Table 2. Standardized mean difference (SMD) of covariates before and after propensity score matching of the Normal-or-mild-AKI and Moderate-to-severe-AKI cohorts

| Characteristic | Before matcing | After matcing |
| --- | --- | --- |
| SMD ≤ 0.1 | 17 | 33 |
| SMD > 0.1 | 22 | 6 |
| Total number of covariates | 39 | 39 |

Supplementary Table 3. Baseline characteristics before propensity score matching of the Non-AKI and Mild-AKI cohorts

|  | **Non-AKI (N=1409)** | **Mild-AKI (N=1175)** | **p-value** | **SMD** | **Missing data (%)** |
| --- | --- | --- | --- | --- | --- |
| Age | **68.00 [59.00, 79.00]** | **71.00 [62.00, 79.00]** | **<0.001** | **0.162** | **0.00** |
| Gender (Female) | **513 (36.41%)** | **376 (32.00%)** | **<0.05** | **0.093** | **0.00** |
| **ICU Score** |  |  |  |  |  |
| SOFA_Score | **2.00 [1.00, 4.00]** | **4.00 [2.00, 6.00]** | **<0.001** | **0.473** | **0.00** |
| AKI_kidigo |  |  |  |  |  |
| 0 | 1409 (100.00%) | 0 (0.00%) |  | <0.001 | 0.00 |
| 1 | 0 (0.00%) | 1175 (100.00%) |  |  |  |
| 2 | 0 (0.00%) | 0 (0.00%) |  |  |  |
| 3 | 0 (0.00%) | 0 (0.00%) |  |  |  |
| **Surgeries and Procedures** |  |  |  |  |  |
| Coronary_Artery_Bypass_Grafting (YES) | **186 (13.20%)** | **264 (22.47%)** | **<0.001** | **0.244** | **0.00** |
| Percutaneous_Coronary_Intervention (YES) | **193 (13.70%)** | **88 (7.49%)** | **<0.001** | **0.203** | **0.00** |
| Continuous_Renal_Replacement_Therapy (YES) | **6 (0.43%)** | **19 (1.62%)** | **<0.01** | **0.119** | **0.00** |
| IABP (YES) | **63 (4.47%)** | **97 (8.26%)** | **<0.001** | **0.155** | **0.00** |
| **Drug Use** |  |  |  |  |  |
| ACEIARB (YES) | 462 (32.79%) | 366 (31.15%) | 0.397 | 0.035 | 0.00 |
| Anticoagulant (YES) | **1125 (79.84%)** | **978 (83.23%)** | **<0.05** | **0.087** | **0.00** |
| Antiplatelet (YES) | **1223 (86.80%)** | **1055 (89.79%)** | **<0.05** | **0.093** | **0.00** |
| B_blocker (YES) | **1020 (72.39%)** | **943 (80.26%)** | **<0.001** | **0.186** | **0.00** |
| Loop_diuretic (YES) | **542 (38.47%)** | **769 (65.45%)** | **<0.001** | **0.561** | **0.00** |
| Positive_inotropic (YES) | **263 (18.67%)** | **159 (13.53%)** | **<0.001** | **0.14** | **0.00** |
| Spironolactone (YES) | 21 (1.49%) | 27 (2.30%) | 0.171 | 0.059 | 0.00 |
| Statin (YES) | **1141 (80.98%)** | **1005 (85.53%)** | **<0.01** | **0.122** | **0.00** |
| Vasopressor (YES) | **503 (35.70%)** | **664 (56.51%)** | **<0.001** | **0.427** | **0.00** |
| **Comorbidities** |  |  |  |  |  |
| HF (YES) | **464 (32.93%)** | **546 (46.47%)** | **<0.001** | **0.279** | **0.00** |
| AFIB (YES) | **112 (7.95%)** | **124 (10.55%)** | **<0.05** | **0.09** | **0.00** |
| Diabetes (YES) | **442 (31.37%)** | **481 (40.94%)** | **<0.001** | **0.2** | **0.00** |
| Renal (YES) | **256 (18.17%)** | **356 (30.30%)** | **<0.001** | **0.286** | **0.00** |
| Liver (YES) | 12 (0.85%) | 12 (1.02%) | 0.809 | 0.018 | 0.00 |
| COPD (YES) | **154 (10.93%)** | **165 (14.04%)** | **<0.05** | **0.094** | **0.00** |
| Stroke (YES) | **77 (5.46%)** | **88 (7.49%)** | **<0.05** | **0.082** | **0.00** |
| Malignancy (YES) | 124 (8.80%) | 124 (10.55%) | 0.15 | 0.059 | 0.00 |
| **Vital signs (1st 24 h)** |  |  |  |  |  |
| MAP | **84.00 [74.00, 96.00]** | **82.00 [72.00, 92.00]** | **<0.001** | **0.13** | **0.00** |
| Heart_Rate | 81.00 [71.00, 93.00] | 81.00 [73.00, 92.00] | 0.502 | 0.048 | 0.00 |
| Temperature | **36.61 [36.39, 36.89]** | **36.56 [36.33, 36.89]** | **<0.05** | **0.046** | **0.00** |
| **Laboratory tests (1st 24 h)** |  |  |  |  |  |
| WBC | **10.80 [8.20, 13.60]** | **11.00 [8.30, 14.90]** | **<0.05** | **0.057** | **0.00** |
| Hemoglobin | **11.60 [9.80, 13.30]** | **10.70 [9.00, 12.60]** | **<0.001** | **0.283** | **0.00** |
| Platelet | **202.00 [156.00, 250.00]** | **187.00 [138.00, 240.50]** | **<0.001** | **0.117** | **0.00** |
| Sodium | **138.00 [135.00, 140.00]** | **137.00 [134.00, 140.00]** | **<0.001** | **0.133** | **0.00** |
| Potassium | **4.20 [3.90, 4.60]** | **4.30 [3.90, 4.80]** | **<0.001** | **0.189** | **0.00** |
| Bicarbonate | **23.00 [21.00, 25.00]** | **23.00 [20.00, 25.00]** | **<0.001** | **0.106** | **0.00** |
| Chloride | 104.00 [101.00, 107.00] | 104.00 [101.00, 107.00] | 0.331 | 0.095 | 0.00 |
| BUN | **17.00 [13.00, 26.00]** | **20.00 [15.00, 33.00]** | **<0.001** | **0.196** | **0.00** |
| Creatinine | **0.90 [0.80, 1.20]** | **1.00 [0.80, 1.60]** | **<0.001** | **0.282** | **0.12** |
| eGFR | **77.02 [55.06, 97.32]** | **65.80 [39.71, 88.27]** | **<0.001** | **0.334** | **0.00** |
| BNP_tag (YES) | 45 (3.19%) | 45 (3.83%) | 0.441 | 0.035 | 0.00 |
| TNT_tag (YES) | **946 (67.14%)** | **648 (55.15%)** | **<0.001** | **0.248** | **0.00** |
| CK_tag (YES) | **652 (46.27%)** | **449 (38.21%)** | **<0.001** | **0.164** | **0.00** |
| Values are presented as mean (standard deviation) or median [Q1, Q3] for continuous variables and number (percentage) for categorical variables. Variables in bold have p-value < 0.05. | | | | | |

Supplementary Table 7. Baseline characteristics after propensity score matching of the Non-AKI and Mild-AKI cohorts

|  | **Non-AKI (N=617)** | **Mild-AKI (N=617)** | **p-value** | **SMD** | **Missing data (%)** |
| --- | --- | --- | --- | --- | --- |
| Age | 70.00 [61.00, 78.00] | 69.00 [61.00, 78.00] | 0.347 | 0.038 | 0.00 |
| Gender (Female) | 221 (35.82%) | 221 (35.82%) | 1 | <0.001 | 0.00 |
| **ICU Score** |  |  |  |  |  |
| SOFA_Score | **4.00 [2.00, 6.00]** | **3.00 [1.00, 5.00]** | **<0.001** | **0.277** | **0.00** |
| AKI_kidigo |  |  |  |  |  |
| 0 | 617 (100.00%) | 0 (0.00%) |  | <0.001 | 0.00 |
| 1 | 0 (0.00%) | 617 (100.00%) |  |  |  |
| 2 | 0 (0.00%) | 0 (0.00%) |  |  |  |
| 3 | 0 (0.00%) | 0 (0.00%) |  |  |  |
| **Surgeries and Procedures** |  |  |  |  |  |
| Coronary_Artery_Bypass_Grafting (YES) | 129 (20.91%) | 118 (19.12%) | 0.477 | 0.045 | 0.00 |
| Percutaneous_Coronary_Intervention (YES) | 51 (8.27%) | 57 (9.24%) | 0.614 | 0.034 | 0.00 |
| Continuous_Renal_Replacement_Therapy (YES) | 4 (0.65%) | 2 (0.32%) | 0.682 | 0.047 | 0.00 |
| IABP (YES) | 36 (5.83%) | 28 (4.54%) | 0.369 | 0.058 | 0.00 |
| **Drug Use** |  |  |  |  |  |
| ACEIARB (YES) | 175 (28.36%) | 194 (31.44%) | 0.263 | 0.067 | 0.00 |
| Anticoagulant (YES) | 495 (80.23%) | 498 (80.71%) | 0.886 | 0.012 | 0.00 |
| Antiplatelet (YES) | 546 (88.49%) | 550 (89.14%) | 0.786 | 0.021 | 0.00 |
| B_blocker (YES) | 487 (78.93%) | 476 (77.15%) | 0.492 | 0.043 | 0.00 |
| Loop_diuretic (YES) | **369 (59.81%)** | **302 (48.95%)** | **<0.001** | **0.219** | **0.00** |
| Positive_inotropic (YES) | 78 (12.64%) | 100 (16.21%) | 0.089 | 0.102 | 0.00 |
| Spironolactone (YES) | 11 (1.78%) | 13 (2.11%) | 0.837 | 0.023 | 0.00 |
| Statin (YES) | 520 (84.28%) | 512 (82.98%) | 0.59 | 0.035 | 0.00 |
| Vasopressor (YES) | **329 (53.32%)** | **269 (43.60%)** | **<0.001** | **0.196** | **0.00** |
| **Comorbidities** |  |  |  |  |  |
| HF (YES) | 246 (39.87%) | 231 (37.44%) | 0.413 | 0.05 | 0.00 |
| AFIB (YES) | 59 (9.56%) | 48 (7.78%) | 0.312 | 0.063 | 0.00 |
| Diabetes (YES) | 232 (37.60%) | 218 (35.33%) | 0.442 | 0.047 | 0.00 |
| Renal (YES) | **153 (24.80%)** | **122 (19.77%)** | **<0.05** | **0.121** | **0.00** |
| Liver (YES) | 8 (1.30%) | 6 (0.97%) | 0.788 | 0.031 | 0.00 |
| COPD (YES) | 77 (12.48%) | 80 (12.97%) | 0.864 | 0.015 | 0.00 |
| Stroke (YES) | 35 (5.67%) | 41 (6.65%) | 0.554 | 0.04 | 0.00 |
| Malignancy (YES) | 56 (9.08%) | 59 (9.56%) | 0.845 | 0.017 | 0.00 |
| **Vital signs (1st 24 h)** |  |  |  |  |  |
| MAP | 82.00 [72.00, 93.00] | 83.00 [73.00, 93.00] | 0.452 | 0.025 | 0.00 |
| Heart_Rate | 81.00 [73.00, 91.00] | 80.00 [73.00, 91.00] | 0.304 | 0.062 | 0.00 |
| Temperature | 36.56 [36.33, 36.83] | 36.61 [36.39, 36.89] | 0.327 | 0.051 | 0.00 |
| **Laboratory tests (1st 24 h)** |  |  |  |  |  |
| WBC | 11.00 [8.40, 14.30] | 10.80 [8.20, 14.00] | 0.299 | 0.064 | 0.00 |
| Hemoglobin | **10.90 [9.40, 12.70]** | **11.40 [9.60, 13.10]** | **<0.01** | **0.157** | **0.00** |
| Platelet | 189.00 [136.00, 242.00] | 192.00 [149.00, 249.00] | 0.093 | 0.093 | 0.00 |
| Sodium | **137.00 [135.00, 139.00]** | **138.00 [135.00, 140.00]** | **<0.01** | **0.118** | **0.00** |
| Potassium | **4.30 [3.90, 4.80]** | **4.20 [3.90, 4.60]** | **<0.05** | **0.125** | **0.00** |
| Bicarbonate | 23.00 [21.00, 25.00] | 23.00 [21.00, 25.00] | 0.15 | 0.074 | 0.00 |
| Chloride | 104.00 [101.00, 107.00] | 104.00 [101.00, 107.00] | 0.437 | 0.027 | 0.00 |
| BUN | **18.00 [14.00, 31.00]** | **17.00 [13.00, 25.00]** | **<0.001** | **0.184** | **0.00** |
| Creatinine | **1.00 [0.80, 1.40]** | **0.90 [0.80, 1.20]** | **<0.01** | **0.204** | **0.16** |
| eGFR | **70.12 [44.62, 91.88]** | **75.23 [54.05, 97.32]** | **<0.001** | **0.216** | **0.00** |
| BNP_tag (YES) | 26 (4.21%) | 22 (3.57%) | 0.659 | 0.034 | 0.00 |
| TNT_tag (YES) | 354 (57.37%) | 376 (60.94%) | 0.224 | 0.073 | 0.00 |
| CK_tag (YES) | 240 (38.90%) | 261 (42.30%) | 0.246 | 0.069 | 0.00 |
| Values are presented as mean (standard deviation) or median [Q1, Q3] for continuous variables and number (percentage) for categorical variables. Variables in bold have p-value < 0.05.  Supplementary Table 4. Baseline characteristics before propensity score matching of the Normal-or-mild-AKI and Moderate-to-severe-AKI cohorts   \|  \| **Normal-or-mild-AKI (N=2584)** \| **Moderate-to-severe-AKI (N=3131)** \| **p-value** \| **SMD** \| **Missing data (%)** \| \| --- \| --- \| --- \| --- \| --- \| --- \| \| Age \| **69.00 [60.00, 79.00]** \| **73.00 [64.00, 81.00]** \| **<0.001** \| **0.218** \| **0.00** \| \| Gender (Female) \| 889 (34.40%) \| 1105 (35.29%) \| 0.501 \| 0.019 \| 0.00 \| \| **ICU Score** \|  \|  \|  \|  \|  \| \| SOFA_Score \| **3.00 [1.00, 5.00]** \| **5.00 [3.00, 8.00]** \| **<0.001** \| **0.538** \| **0.00** \| \| AKI_kidigo \|  \|  \|  \|  \|  \| \| 0 \| **1409 (54.53%)** \| **0 (0.00%)** \| **<0.001** \| **3.88** \| **0.00** \| \| 1 \| **1175 (45.47%)** \| **0 (0.00%)** \|  \|  \|  \| \| 2 \| **0 (0.00%)** \| **2065 (65.95%)** \|  \|  \|  \| \| 3 \| **0 (0.00%)** \| **1066 (34.05%)** \|  \|  \|  \| \| **Surgeries and Procedures** \|  \|  \|  \|  \|  \| \| Coronary_Artery_Bypass_Grafting (YES) \| 450 (17.41%) \| 530 (16.93%) \| 0.652 \| 0.013 \| 0.00 \| \| Percutaneous_Coronary_Intervention (YES) \| **281 (10.87%)** \| **217 (6.93%)** \| **<0.001** \| **0.139** \| **0.00** \| \| Continuous_Renal_Replacement_Therapy (YES) \| **25 (0.97%)** \| **130 (4.15%)** \| **<0.001** \| **0.203** \| **0.00** \| \| IABP (YES) \| **160 (6.19%)** \| **442 (14.12%)** \| **<0.001** \| **0.265** \| **0.00** \| \| **Drug Use** \|  \|  \|  \|  \|  \| \| ACEIARB (YES) \| 828 (32.04%) \| 1054 (33.66%) \| 0.205 \| 0.034 \| 0.00 \| \| Anticoagulant (YES) \| **2103 (81.39%)** \| **2821 (90.10%)** \| **<0.001** \| **0.251** \| **0.00** \| \| Antiplatelet (YES) \| **2278 (88.16%)** \| **2845 (90.87%)** \| **<0.001** \| **0.088** \| **0.00** \| \| B_blocker (YES) \| **1963 (75.97%)** \| **2489 (79.50%)** \| **<0.01** \| **0.085** \| **0.00** \| \| Loop_diuretic (YES) \| **1311 (50.74%)** \| **2251 (71.89%)** \| **<0.001** \| **0.445** \| **0.00** \| \| Positive_inotropic (YES) \| 422 (16.33%) \| 554 (17.69%) \| 0.184 \| 0.036 \| 0.00 \| \| Spironolactone (YES) \| 48 (1.86%) \| 78 (2.49%) \| 0.125 \| 0.043 \| 0.00 \| \| Statin (YES) \| **2146 (83.05%)** \| **2743 (87.61%)** \| **<0.001** \| **0.129** \| **0.00** \| \| Vasopressor (YES) \| **1167 (45.16%)** \| **2146 (68.54%)** \| **<0.001** \| **0.486** \| **0.00** \| \| **Comorbidities** \|  \|  \|  \|  \|  \| \| HF (YES) \| **1010 (39.09%)** \| **1739 (55.54%)** \| **<0.001** \| **0.334** \| **0.00** \| \| AFIB (YES) \| **236 (9.13%)** \| **492 (15.71%)** \| **<0.001** \| **0.201** \| **0.00** \| \| Diabetes (YES) \| **923 (35.72%)** \| **1303 (41.62%)** \| **<0.001** \| **0.121** \| **0.00** \| \| Renal_Disease (YES) \| **612 (23.68%)** \| **1003 (32.03%)** \| **<0.001** \| **0.187** \| **0.00** \| \| Liver_Disease (YES) \| **24 (0.93%)** \| **66 (2.11%)** \| **<0.001** \| **0.097** \| **0.00** \| \| COPD (YES) \| **319 (12.35%)** \| **489 (15.62%)** \| **<0.001** \| **0.094** \| **0.00** \| \| Stroke (YES) \| **165 (6.39%)** \| **373 (11.91%)** \| **<0.001** \| **0.193** \| **0.00** \| \| Malignancy (YES) \| 248 (9.60%) \| 345 (11.02%) \| 0.087 \| 0.047 \| 0.00 \| \| **Vital signs (1st 24 h)** \|  \|  \|  \|  \|  \| \| MAP \| **83.00 [73.00, 94.00]** \| **81.00 [71.00, 94.00]** \| **<0.001** \| **0.045** \| **0.00** \| \| Heart_Rate \| **81.00 [72.00, 92.00]** \| **84.00 [74.00, 97.00]** \| **<0.001** \| **0.148** \| **0.00** \| \| Temperature \| **36.61 [36.39, 36.89]** \| **36.61 [36.33, 36.94]** \| **<0.05** \| **0.037** \| **0.00** \| \| **Laboratory tests (1st 24 h)** \|  \|  \|  \|  \|  \| \| WBC \| **10.90 [8.30, 14.12]** \| **12.20 [9.10, 16.30]** \| **<0.001** \| **0.152** \| **0.00** \| \| Hemoglobin \| **11.20 [9.40, 13.00]** \| **10.90 [9.10, 12.70]** \| **<0.001** \| **0.098** \| **0.00** \| \| Platelet \| 195.00 [146.00, 246.25] \| 192.00 [145.00, 251.00] \| 0.88 \| 0.016 \| 0.00 \| \| Sodium \| 137.00 [135.00, 140.00] \| 137.00 [134.00, 140.00] \| 0.941 \| 0.026 \| 0.00 \| \| Potassium \| 4.20 [3.90, 4.70] \| 4.30 [3.90, 4.80] \| 0.238 \| 0.033 \| 0.00 \| \| Bicarbonate \| **23.00 [21.00, 25.00]** \| **22.00 [20.00, 25.00]** \| **<0.001** \| **0.128** \| **0.00** \| \| Chloride \| **104.00 [101.00, 107.00]** \| **104.00 [100.00, 107.00]** \| **<0.001** \| **0.106** \| **0.00** \| \| BUN \| **18.00 [14.00, 29.00]** \| **23.00 [16.00, 36.00]** \| **<0.001** \| **0.217** \| **0.00** \| \| Creatinine \| **1.00 [0.80, 1.40]** \| **1.10 [0.90, 1.70]** \| **<0.001** \| **0.215** \| **0.07** \| \| eGFR \| **72.08 [46.56, 95.29]** \| **57.75 [35.26, 81.81]** \| **<0.001** \| **0.312** \| **0.00** \| \| BNP_tag (YES) \| **90 (3.48%)** \| **170 (5.43%)** \| **<0.001** \| **0.094** \| **0.00** \| \| TNT_tag (YES) \| **1594 (61.69%)** \| **2093 (66.85%)** \| **<0.001** \| **0.108** \| **0.00** \| \| CK_tag (YES) \| **1101 (42.61%)** \| **1527 (48.77%)** \| **<0.001** \| **0.124** \| **0.00** \| \| Values are presented as mean (standard deviation) or median [Q1, Q3] for continuous variables and number (percentage) for categorical variables. Variables in bold have p-value < 0.05. \| \| \| \| \| \| | | | | | |

Supplementary Table 5. Multivariate logistic model adjusted with all covariates for 28-day mortality of the Non-AKI and Mild-AKI cohorts

| **Characteristic** | **OR**^1^ | **95% CI**^1^ | **p-value** |
| --- | --- | --- | --- |
| Group |  |  |  |
| Non-AKI | 1.00 | Reference |  |
| Mild-AKI | 0.94 | 0.64, 1.36 | 0.736 |
| Age | 1.06 | 1.04, 1.08 | <0.001 |
| Gender |  |  |  |
| Female | 1.00 | Reference |  |
| Male | 1.09 | 0.76, 1.59 | 0.633 |
| SOFA_Score | 1.25 | 1.16, 1.34 | <0.001 |
| Coronary_Artery_Bypass_Grafting |  |  |  |
| YES | 1.00 | Reference |  |
| NO | 14.9 | 4.00, 102 | <0.001 |
| Percutaneous_Coronary_Intervention |  |  |  |
| YES | 1.00 | Reference |  |
| NO | 0.88 | 0.47, 1.71 | 0.694 |
| Continuous_Renal_Replacement_Therapy |  |  |  |
| YES | 1.00 | Reference |  |
| NO | 0.61 | 0.16, 2.71 | 0.493 |
| IABP |  |  |  |
| YES | 1.00 | Reference |  |
| NO | 0.45 | 0.23, 0.93 | 0.027 |
| ACEIARB |  |  |  |
| YES | 1.00 | Reference |  |
| NO | 1.60 | 0.96, 2.78 | 0.083 |
| Anticoagulant |  |  |  |
| YES | 1.00 | Reference |  |
| NO | 0.86 | 0.52, 1.38 | 0.537 |
| Antiplatelet |  |  |  |
| YES | 1.00 | Reference |  |
| NO | 1.58 | 1.00, 2.50 | 0.05 |
| B_blocker |  |  |  |
| YES | 1.00 | Reference |  |
| NO | 2.50 | 1.68, 3.72 | <0.001 |
| Loop_diuretic |  |  |  |
| YES | 1.00 | Reference |  |
| NO | 0.83 | 0.55, 1.25 | 0.386 |
| Positive_inotropic |  |  |  |
| YES | 1.00 | Reference |  |
| NO | 0.96 | 0.54, 1.82 | 0.907 |
| Spironolactone |  |  |  |
| YES | 1.00 | Reference |  |
| NO | 1.25 | 0.37, 5.96 | 0.748 |
| Statin |  |  |  |
| YES | 1.00 | Reference |  |
| NO | 1.93 | 1.26, 2.95 | 0.003 |
| Vasopressor |  |  |  |
| YES | 1.00 | Reference |  |
| NO | 0.78 | 0.50, 1.20 | 0.248 |
| HF |  |  |  |
| YES | 1.00 | Reference |  |
| NO | 1.23 | 0.83, 1.84 | 0.308 |
| AFIB |  |  |  |
| YES | 1.00 | Reference |  |
| NO | 1.20 | 0.66, 2.25 | 0.565 |
| Diabetes |  |  |  |
| YES | 1.00 | Reference |  |
| NO | 1.10 | 0.75, 1.62 | 0.639 |
| Renal |  |  |  |
| YES | 1.00 | Reference |  |
| NO | 1.00 | 0.64, 1.57 | 0.994 |
| Liver |  |  |  |
| YES | 1.00 | Reference |  |
| NO | 0.30 | 0.10, 1.13 | 0.051 |
| COPD |  |  |  |
| YES | 1.00 | Reference |  |
| NO | 0.85 | 0.54, 1.36 | 0.48 |
| Stroke |  |  |  |
| YES | 1.00 | Reference |  |
| NO | 0.24 | 0.14, 0.40 | <0.001 |
| Malignancy |  |  |  |
| YES | 1.00 | Reference |  |
| NO | 0.43 | 0.27, 0.67 | <0.001 |
| MAP | 0.99 | 0.98, 1.00 | 0.024 |
| Heart_Rate | 1.01 | 1.01, 1.02 | 0.001 |
| Temperature | 0.65 | 0.52, 0.82 | <0.001 |
| WBC | 1.00 | 0.99, 1.01 | 0.929 |
| Hemoglobin | 1.03 | 0.94, 1.12 | 0.569 |
| Platelet | 1.00 | 1.00, 1.00 | 0.055 |
| Sodium | 1.07 | 1.03, 1.12 | 0.002 |
| Potassium | 0.99 | 0.78, 1.25 | 0.941 |
| Bicarbonate | 0.92 | 0.87, 0.96 | <0.001 |
| Chloride | 0.92 | 0.89, 0.96 | <0.001 |
| BUN | 1.00 | 0.99, 1.01 | 0.741 |
| eGFR | 1.00 | 0.99, 1.01 | 0.856 |
| BNP_tag |  |  |  |
| YES | 1.00 | Reference |  |
| NO | 0.88 | 0.42, 1.99 | 0.756 |
| TNT_tag |  |  |  |
| YES | 1.00 | Reference |  |
| NO | 1.18 | 0.74, 1.86 | 0.486 |
| CK_tag |  |  |  |
| YES | 1.00 | Reference |  |
| NO | 0.80 | 0.53, 1.21 | 0.292 |
| ^1^OR = Odds Ratio, CI = Confidence Interval  Supplementary Table 6. Multivariate logistic model adjusted with all covariates for 28-day mortality of the Normal-or-mild-AKI and Moderate-to-severe-AKI cohorts   \| **Characteristic** \| **OR**^1^ \| **95% CI**^1^ \| **p-value** \| \| --- \| --- \| --- \| --- \| \| Group \|  \|  \|  \| \| Normal-or-mild-AKI \| 1.00 \| Reference \|  \| \| Moderate-to-severe-AKI \| 2.01 \| 1.63, 2.48 \| <0.001 \| \| Age \| 1.05 \| 1.04, 1.06 \| <0.001 \| \| Gender \|  \|  \|  \| \| Female \| 1.00 \| Reference \|  \| \| Male \| 1.00 \| 0.83, 1.21 \| 0.982 \| \| SOFA_Score \| 1.15 \| 1.12, 1.19 \| <0.001 \| \| Coronary_Artery_Bypass_Grafting \|  \|  \|  \| \| YES \| 1.00 \| Reference \|  \| \| NO \| 5.82 \| 3.50, 10.2 \| <0.001 \| \| Percutaneous_Coronary_Intervention \|  \|  \|  \| \| YES \| 1.00 \| Reference \|  \| \| NO \| 0.74 \| 0.53, 1.03 \| 0.071 \| \| Continuous_Renal_Replacement_Therapy \|  \|  \|  \| \| YES \| 1.00 \| Reference \|  \| \| NO \| 0.87 \| 0.55, 1.37 \| 0.545 \| \| IABP \|  \|  \|  \| \| YES \| 1.00 \| Reference \|  \| \| NO \| 0.90 \| 0.67, 1.20 \| 0.455 \| \| ACEIARB \|  \|  \|  \| \| YES \| 1.00 \| Reference \|  \| \| NO \| 2.13 \| 1.69, 2.71 \| <0.001 \| \| Anticoagulant \|  \|  \|  \| \| YES \| 1.00 \| Reference \|  \| \| NO \| 0.86 \| 0.62, 1.17 \| 0.341 \| \| Antiplatelet \|  \|  \|  \| \| YES \| 1.00 \| Reference \|  \| \| NO \| 1.14 \| 0.86, 1.52 \| 0.355 \| \| B_blocker \|  \|  \|  \| \| YES \| 1.00 \| Reference \|  \| \| NO \| 2.76 \| 2.25, 3.38 \| <0.001 \| \| Loop_diuretic \|  \|  \|  \| \| YES \| 1.00 \| Reference \|  \| \| NO \| 0.89 \| 0.72, 1.10 \| 0.277 \| \| Positive_inotropic \|  \|  \|  \| \| YES \| 1.00 \| Reference \|  \| \| NO \| 0.70 \| 0.54, 0.91 \| 0.006 \| \| Spironolactone \|  \|  \|  \| \| YES \| 1.00 \| Reference \|  \| \| NO \| 0.66 \| 0.39, 1.16 \| 0.131 \| \| Statin \|  \|  \|  \| \| YES \| 1.00 \| Reference \|  \| \| NO \| 1.64 \| 1.28, 2.10 \| <0.001 \| \| Vasopressor \|  \|  \|  \| \| YES \| 1.00 \| Reference \|  \| \| NO \| 0.62 \| 0.50, 0.78 \| <0.001 \| \| HF \|  \|  \|  \| \| YES \| 1.00 \| Reference \|  \| \| NO \| 0.90 \| 0.74, 1.11 \| 0.321 \| \| AFIB \|  \|  \|  \| \| YES \| 1.00 \| Reference \|  \| \| NO \| 0.88 \| 0.68, 1.15 \| 0.342 \| \| Diabetes \|  \|  \|  \| \| YES \| 1.00 \| Reference \|  \| \| NO \| 0.99 \| 0.82, 1.19 \| 0.888 \| \| Renal_Disease \|  \|  \|  \| \| YES \| 1.00 \| Reference \|  \| \| NO \| 1.27 \| 1.01, 1.60 \| 0.041 \| \| Liver_Disease \|  \|  \|  \| \| YES \| 1.00 \| Reference \|  \| \| NO \| 0.54 \| 0.31, 0.98 \| 0.039 \| \| COPD \|  \|  \|  \| \| YES \| 1.00 \| Reference \|  \| \| NO \| 0.65 \| 0.52, 0.81 \| <0.001 \| \| Stroke \|  \|  \|  \| \| YES \| 1.00 \| Reference \|  \| \| NO \| 0.41 \| 0.32, 0.54 \| <0.001 \| \| Malignancy \|  \|  \|  \| \| YES \| 1.00 \| Reference \|  \| \| NO \| 0.61 \| 0.48, 0.78 \| <0.001 \| \| MAP \| 1.00 \| 0.99, 1.00 \| 0.274 \| \| Heart_Rate \| 1.01 \| 1.00, 1.01 \| <0.001 \| \| Temperature \| 0.82 \| 0.74, 0.91 \| <0.001 \| \| WBC \| 1.00 \| 0.99, 1.01 \| 0.629 \| \| Hemoglobin \| 1.0 \| 0.95, 1.04 \| 0.815 \| \| Platelet \| 1.00 \| 1.00, 1.00 \| 0.003 \| \| Sodium \| 1.09 \| 1.06, 1.12 \| <0.001 \| \| Potassium \| 0.92 \| 0.82, 1.04 \| 0.193 \| \| Bicarbonate \| 0.93 \| 0.91, 0.95 \| <0.001 \| \| Chloride \| 0.92 \| 0.90, 0.94 \| <0.001 \| \| BUN \| 1.01 \| 1.00, 1.01 \| 0.026 \| \| eGFR \| 1.00 \| 0.99, 1.00 \| 0.364 \| \| BNP_tag \|  \|  \|  \| \| YES \| 1.00 \| Reference \|  \| \| NO \| 1.18 \| 0.82, 1.71 \| 0.383 \| \| TNT_tag \|  \|  \|  \| \| YES \| 1.00 \| Reference \|  \| \| NO \| 0.99 \| 0.77, 1.27 \| 0.948 \| \| CK_tag \|  \|  \|  \| \| YES \| 1.00 \| Reference \|  \| \| NO \| 0.76 \| 0.61, 0.93 \| 0.01 \| \| ^1^OR = Odds Ratio, CI = Confidence Interval \| \| \| \| | | | |

Supplementary Table 7. Multivariate logistic model adjusted with unbalanced covariates for 28-day mortality of the Non-AKI and Mild-AKI cohorts

| **Characteristic** | **OR**^1^ | **95% CI**^1^ | **p-value** |
| --- | --- | --- | --- |
| Group |  |  |  |
| Non-AKI | 1.00 | Reference |  |
| Mild-AKI | 0.90 | 0.69, 1.17 | 0.444 |
| Age | 1.06 | 1.05, 1.07 | <0.001 |
| Gender |  |  |  |
| Female | 1.00 | Reference |  |
| Male | 1.06 | 0.80, 1.40 | 0.681 |
| SOFA_Score | 1.26 | 1.20, 1.33 | <0.001 |
| Coronary_Artery_Bypass_Grafting |  |  |  |
| YES | 1.00 | Reference |  |
| NO | 8.04 | 3.47, 22.3 | <0.001 |
| Percutaneous_Coronary_Intervention |  |  |  |
| YES | 1.00 | Reference |  |
| NO | 0.87 | 0.55, 1.41 | 0.559 |
| Continuous_Renal_Replacement_Therapy |  |  |  |
| YES | 1.00 | Reference |  |
| NO | 0.95 | 0.30, 3.37 | 0.932 |
| IABP |  |  |  |
| YES | 1.00 | Reference |  |
| NO | 0.54 | 0.32, 0.95 | 0.029 |
| ACEIARB |  |  |  |
| YES | 1.00 | Reference |  |
| NO | 1.46 | 1.00, 2.18 | 0.057 |
| Anticoagulant |  |  |  |
| YES | 1.00 | Reference |  |
| NO | 0.83 | 0.57, 1.19 | 0.316 |
| Antiplatelet |  |  |  |
| YES | 1.00 | Reference |  |
| NO | 1.65 | 1.17, 2.32 | 0.004 |
| B_blocker |  |  |  |
| YES | 1.00 | Reference |  |
| NO | 2.24 | 1.66, 3.02 | <0.001 |
| Loop_diuretic |  |  |  |
| YES | 1.00 | Reference |  |
| NO | 0.79 | 0.59, 1.08 | 0.14 |
| Positive_inotropic |  |  |  |
| YES | 1.00 | Reference |  |
| NO | 0.85 | 0.55, 1.34 | 0.469 |
| Spironolactone |  |  |  |
| YES | 1.00 | Reference |  |
| NO | 1.14 | 0.45, 3.51 | 0.797 |
| Statin |  |  |  |
| YES | 1.00 | Reference |  |
| NO | 1.95 | 1.42, 2.69 | <0.001 |
| Vasopressor |  |  |  |
| YES | 1.00 | Reference |  |
| NO | 0.77 | 0.56, 1.07 | 0.114 |
| HF |  |  |  |
| YES | 1.00 | Reference |  |
| NO | 1.23 | 0.91, 1.66 | 0.176 |
| AFIB |  |  |  |
| YES | 1.00 | Reference |  |
| NO | 1.23 | 0.78, 1.99 | 0.381 |
| Diabetes |  |  |  |
| YES | 1.00 | Reference |  |
| NO | 1.08 | 0.81, 1.44 | 0.611 |
| Renal |  |  |  |
| YES | 1.00 | Reference |  |
| NO | 1.01 | 0.72, 1.42 | 0.943 |
| Liver |  |  |  |
| YES | 1.00 | Reference |  |
| NO | 0.30 | 0.13, 0.76 | 0.007 |
| COPD |  |  |  |
| YES | 1.00 | Reference |  |
| NO | 0.86 | 0.61, 1.22 | 0.382 |
| Stroke |  |  |  |
| YES | 1.00 | Reference |  |
| NO | 0.23 | 0.16, 0.35 | <0.001 |
| Malignancy |  |  |  |
| YES | 1.00 | Reference |  |
| NO | 0.41 | 0.30, 0.58 | <0.001 |
| MAP | 0.99 | 0.98, 1.00 | 0.027 |
| Heart_Rate | 1.01 | 1.01, 1.02 | <0.001 |
| Temperature | 0.63 | 0.53, 0.75 | <0.001 |
| WBC | 1.00 | 0.99, 1.01 | 0.598 |
| Hemoglobin | 1.04 | 0.98, 1.12 | 0.195 |
| Platelet | 1.00 | 1.00, 1.00 | 0.011 |
| Sodium | 1.07 | 1.04, 1.11 | <0.001 |
| Potassium | 0.97 | 0.80, 1.16 | 0.711 |
| Bicarbonate | 0.92 | 0.89, 0.95 | <0.001 |
| Chloride | 0.93 | 0.91, 0.95 | <0.001 |
| BUN | 1.00 | 0.99, 1.01 | 0.96 |
| eGFR | 1.00 | 0.99, 1.00 | 0.625 |
| BNP_tag |  |  |  |
| YES | 1.00 | Reference |  |
| NO | 0.86 | 0.49, 1.57 | 0.617 |
| TNT_tag |  |  |  |
| YES | 1.00 | Reference |  |
| NO | 1.07 | 0.75, 1.51 | 0.71 |
| CK_tag |  |  |  |
| YES | 1.00 | Reference |  |
| NO | 0.88 | 0.65, 1.20 | 0.417 |
| ^1^OR = Odds Ratio, CI = Confidence Interval  Supplementary Table 8. Multivariate logistic model adjusted with unbalanced covariates for 28-day mortality of the Normal-or-mild-AKI and Moderate-to-severe-AKI cohorts   \| **Characteristic** \| **OR**^1^ \| **95% CI**^1^ \| **p-value** \| \| --- \| --- \| --- \| --- \| \| Group \|  \|  \|  \| \| Normal-or-mild-AKI \| 1.00 \| Reference \|  \| \| Moderate-to-severe-AKI \| 1.87 \| 1.62, 2.15 \| <0.001 \| \| Age \| 1.05 \| 1.05, 1.06 \| <0.001 \| \| Gender \|  \|  \|  \| \| Female \| 1.00 \| Reference \|  \| \| Male \| 0.95 \| 0.82, 1.10 \| 0.468 \| \| SOFA_Score \| 1.19 \| 1.16, 1.22 \| <0.001 \| \| Coronary_Artery_Bypass_Grafting \|  \|  \|  \| \| YES \| 1.00 \| Reference \|  \| \| NO \| 5.77 \| 3.88, 8.87 \| <0.001 \| \| Percutaneous_Coronary_Intervention \|  \|  \|  \| \| YES \| 1.00 \| Reference \|  \| \| NO \| 0.71 \| 0.55, 0.92 \| 0.009 \| \| Continuous_Renal_Replacement_Therapy \|  \|  \|  \| \| YES \| 1.00 \| Reference \|  \| \| NO \| 0.74 \| 0.51, 1.08 \| 0.121 \| \| IABP \|  \|  \|  \| \| YES \| 1.00 \| Reference \|  \| \| NO \| 0.79 \| 0.63, 0.99 \| 0.037 \| \| ACEIARB \|  \|  \|  \| \| YES \| 1.00 \| Reference \|  \| \| NO \| 1.94 \| 1.61, 2.33 \| <0.001 \| \| Anticoagulant \|  \|  \|  \| \| YES \| 1.00 \| Reference \|  \| \| NO \| 0.88 \| 0.69, 1.12 \| 0.301 \| \| Antiplatelet \|  \|  \|  \| \| YES \| 1.00 \| Reference \|  \| \| NO \| 1.22 \| 0.99, 1.51 \| 0.066 \| \| B_blocker \|  \|  \|  \| \| YES \| 1.00 \| Reference \|  \| \| NO \| 2.63 \| 2.25, 3.07 \| <0.001 \| \| Loop_diuretic \|  \|  \|  \| \| YES \| 1.00 \| Reference \|  \| \| NO \| 0.86 \| 0.73, 1.00 \| 0.054 \| \| Positive_inotropic \|  \|  \|  \| \| YES \| 1.00 \| Reference \|  \| \| NO \| 0.67 \| 0.55, 0.82 \| <0.001 \| \| Spironolactone \|  \|  \|  \| \| YES \| 1.00 \| Reference \|  \| \| NO \| 0.69 \| 0.46, 1.06 \| 0.082 \| \| Statin \|  \|  \|  \| \| YES \| 1.00 \| Reference \|  \| \| NO \| 1.64 \| 1.36, 1.99 \| <0.001 \| \| Vasopressor \|  \|  \|  \| \| YES \| 1.00 \| Reference \|  \| \| NO \| 0.65 \| 0.55, 0.77 \| <0.001 \| \| HF \|  \|  \|  \| \| YES \| 1.00 \| Reference \|  \| \| NO \| 1.00 \| 0.86, 1.17 \| 0.975 \| \| AFIB \|  \|  \|  \| \| YES \| 1.00 \| Reference \|  \| \| NO \| 0.95 \| 0.77, 1.16 \| 0.596 \| \| Diabetes \|  \|  \|  \| \| YES \| 1.00 \| Reference \|  \| \| NO \| 0.97 \| 0.84, 1.12 \| 0.674 \| \| Renal_Disease \|  \|  \|  \| \| YES \| 1.00 \| Reference \|  \| \| NO \| 1.17 \| 0.98, 1.39 \| 0.083 \| \| Liver_Disease \|  \|  \|  \| \| YES \| 1.00 \| Reference \|  \| \| NO \| 0.43 \| 0.27, 0.69 \| <0.001 \| \| COPD \|  \|  \|  \| \| YES \| 1.00 \| Reference \|  \| \| NO \| 0.66 \| 0.55, 0.78 \| <0.001 \| \| Stroke \|  \|  \|  \| \| YES \| 1.00 \| Reference \|  \| \| NO \| 0.39 \| 0.32, 0.47 \| <0.001 \| \| Malignancy \|  \|  \|  \| \| YES \| 1.00 \| Reference \|  \| \| NO \| 0.58 \| 0.48, 0.70 \| <0.001 \| \| MAP \| 1.00 \| 0.99, 1.00 \| 0.291 \| \| Heart_Rate \| 1.01 \| 1.01, 1.01 \| <0.001 \| \| Temperature \| 0.82 \| 0.76, 0.90 \| <0.001 \| \| WBC \| 1.00 \| 0.99, 1.01 \| 0.725 \| \| Hemoglobin \| 0.99 \| 0.96, 1.02 \| 0.564 \| \| Platelet \| 1.00 \| 1.00, 1.00 \| <0.001 \| \| Sodium \| 1.09 \| 1.07, 1.11 \| <0.001 \| \| Potassium \| 0.92 \| 0.84, 1.01 \| 0.08 \| \| Bicarbonate \| 0.93 \| 0.91, 0.94 \| <0.001 \| \| Chloride \| 0.91 \| 0.90, 0.93 \| <0.001 \| \| BUN \| 1.00 \| 1.00, 1.01 \| 0.018 \| \| eGFR \| 1.00 \| 1.00, 1.00 \| 0.849 \| \| BNP_tag \|  \|  \|  \| \| YES \| 1.00 \| Reference \|  \| \| NO \| 1.35 \| 1.01, 1.81 \| 0.045 \| \| TNT_tag \|  \|  \|  \| \| YES \| 1.00 \| Reference \|  \| \| NO \| 0.98 \| 0.81, 1.19 \| 0.829 \| \| CK_tag \|  \|  \|  \| \| YES \| 1.00 \| Reference \|  \| \| NO \| 0.74 \| 0.63, 0.87 \| <0.001 \| \| ^1^OR = Odds Ratio, CI = Confidence Interval \| \| \| \| | | | |

Supplementary Table 9. Survey-weighted generalised linear model adjusted with all covariates and IPTW for 28-day mortality of the Non-AKI and Mild-AKI cohorts

| **Characteristic** | **OR**^1^ | **95% CI**^1^ | **p-value** |
| --- | --- | --- | --- |
| Group |  |  |  |
| Non-AKI | 1.00 | Reference |  |
| Mild-AKI | 0.90 | 0.62, 1.31 | 0.588 |
| Age | 1.06 | 1.04, 1.08 | <0.001 |
| Gender |  |  |  |
| Female | 1.00 | Reference |  |
| Male | 1.06 | 0.71, 1.57 | 0.773 |
| SOFA_Score | 1.26 | 1.17, 1.37 | <0.001 |
| Coronary_Artery_Bypass_Grafting |  |  |  |
| YES | 1.00 | Reference |  |
| NO | 8.04 | 1.87, 34.5 | 0.005 |
| Percutaneous_Coronary_Intervention |  |  |  |
| YES | 1.00 | Reference |  |
| NO | 0.87 | 0.44, 1.72 | 0.686 |
| Continuous_Renal_Replacement_Therapy |  |  |  |
| YES | 1.00 | Reference |  |
| NO | 0.95 | 0.16, 5.57 | 0.954 |
| IABP |  |  |  |
| YES | 1.00 | Reference |  |
| NO | 0.54 | 0.30, 0.97 | 0.04 |
| ACEIARB |  |  |  |
| YES | 1.00 | Reference |  |
| NO | 1.46 | 0.83, 2.58 | 0.188 |
| Anticoagulant |  |  |  |
| YES | 1.00 | Reference |  |
| NO | 0.83 | 0.52, 1.31 | 0.416 |
| Antiplatelet |  |  |  |
| YES | 1.00 | Reference |  |
| NO | 1.65 | 1.01, 2.69 | 0.045 |
| B_blocker |  |  |  |
| YES | 1.00 | Reference |  |
| NO | 2.24 | 1.52, 3.31 | <0.001 |
| Loop_diuretic |  |  |  |
| YES | 1.00 | Reference |  |
| NO | 0.79 | 0.51, 1.23 | 0.301 |
| Positive_inotropic |  |  |  |
| YES | 1.00 | Reference |  |
| NO | 0.85 | 0.46, 1.57 | 0.604 |
| Spironolactone |  |  |  |
| YES | 1.00 | Reference |  |
| NO | 1.14 | 0.26, 5.05 | 0.861 |
| Statin |  |  |  |
| YES | 1.00 | Reference |  |
| NO | 1.95 | 1.26, 3.04 | 0.003 |
| Vasopressor |  |  |  |
| YES | 1.00 | Reference |  |
| NO | 0.77 | 0.47, 1.25 | 0.294 |
| HF |  |  |  |
| YES | 1.00 | Reference |  |
| NO | 1.23 | 0.80, 1.89 | 0.343 |
| AFIB |  |  |  |
| YES | 1.00 | Reference |  |
| NO | 1.23 | 0.64, 2.38 | 0.533 |
| Diabetes |  |  |  |
| YES | 1.00 | Reference |  |
| NO | 1.08 | 0.72, 1.60 | 0.712 |
| Renal |  |  |  |
| YES | 1.00 | Reference |  |
| NO | 1.01 | 0.62, 1.65 | 0.961 |
| Liver |  |  |  |
| YES | 1.00 | Reference |  |
| NO | 0.30 | 0.08, 1.11 | 0.071 |
| COPD |  |  |  |
| YES | 1.00 | Reference |  |
| NO | 0.86 | 0.53, 1.38 | 0.525 |
| Stroke |  |  |  |
| YES | 1.00 | Reference |  |
| NO | 0.23 | 0.13, 0.42 | <0.001 |
| Malignancy |  |  |  |
| YES | 1.00 | Reference |  |
| NO | 0.41 | 0.24, 0.71 | 0.001 |
| MAP | 0.99 | 0.98, 1.00 | 0.092 |
| Heart_Rate | 1.01 | 1.00, 1.02 | 0.005 |
| Temperature | 0.63 | 0.48, 0.83 | <0.001 |
| WBC | 1.00 | 0.99, 1.02 | 0.667 |
| Hemoglobin | 1.04 | 0.95, 1.15 | 0.371 |
| Platelet | 1.00 | 1.00, 1.00 | 0.082 |
| Sodium | 1.07 | 1.02, 1.12 | 0.005 |
| Potassium | 0.97 | 0.76, 1.23 | 0.78 |
| Bicarbonate | 0.92 | 0.87, 0.97 | 0.001 |
| Chloride | 0.93 | 0.90, 0.96 | <0.001 |
| BUN | 1.00 | 0.99, 1.01 | 0.974 |
| eGFR | 1.00 | 0.99, 1.01 | 0.741 |
| BNP_tag |  |  |  |
| YES | 1.00 | Reference |  |
| NO | 0.86 | 0.39, 1.90 | 0.715 |
| TNT_tag |  |  |  |
| YES | 1.00 | Reference |  |
| NO | 1.07 | 0.67, 1.71 | 0.784 |
| CK_tag |  |  |  |
| YES | 1.00 | Reference |  |
| NO | 0.88 | 0.56, 1.38 | 0.578 |
| ^1^OR = Odds Ratio, CI = Confidence Interval  Supplementary Table 10. Survey-weighted generalised linear model adjusted with all covariates and IPTW for 28-day mortality of the Normal-or-mild-AKI and Moderate-to-severe-AKI cohorts   \| **Characteristic** \| **OR**^1^ \| **95% CI**^1^ \| **p-value** \| \| --- \| --- \| --- \| --- \| \| Group \|  \|  \|  \| \| Normal-or-mild-AKI \| 1.00 \| Reference \|  \| \| Moderate-to-severe-AKI \| 1.87 \| 1.49, 2.33 \| <0.001 \| \| Age \| 1.05 \| 1.04, 1.06 \| <0.001 \| \| Gender \|  \|  \|  \| \| Female \| 1.00 \| Reference \|  \| \| Male \| 0.95 \| 0.77, 1.17 \| 0.621 \| \| SOFA_Score \| 1.19 \| 1.15, 1.24 \| <0.001 \| \| Coronary_Artery_Bypass_Grafting \|  \|  \|  \| \| YES \| 1.00 \| Reference \|  \| \| NO \| 5.77 \| 3.02, 11.0 \| <0.001 \| \| Percutaneous_Coronary_Intervention \|  \|  \|  \| \| YES \| 1.00 \| Reference \|  \| \| NO \| 0.71 \| 0.49, 1.03 \| 0.068 \| \| Continuous_Renal_Replacement_Therapy \|  \|  \|  \| \| YES \| 1.00 \| Reference \|  \| \| NO \| 0.74 \| 0.44, 1.26 \| 0.271 \| \| IABP \|  \|  \|  \| \| YES \| 1.00 \| Reference \|  \| \| NO \| 0.79 \| 0.57, 1.09 \| 0.148 \| \| ACEIARB \|  \|  \|  \| \| YES \| 1.00 \| Reference \|  \| \| NO \| 1.94 \| 1.47, 2.55 \| <0.001 \| \| Anticoagulant \|  \|  \|  \| \| YES \| 1.00 \| Reference \|  \| \| NO \| 0.88 \| 0.64, 1.21 \| 0.432 \| \| Antiplatelet \|  \|  \|  \| \| YES \| 1.00 \| Reference \|  \| \| NO \| 1.22 \| 0.88, 1.69 \| 0.228 \| \| B_blocker \|  \|  \|  \| \| YES \| 1.00 \| Reference \|  \| \| NO \| 2.63 \| 2.12, 3.26 \| <0.001 \| \| Loop_diuretic \|  \|  \|  \| \| YES \| 1.00 \| Reference \|  \| \| NO \| 0.86 \| 0.68, 1.08 \| 0.182 \| \| Positive_inotropic \|  \|  \|  \| \| YES \| 1.00 \| Reference \|  \| \| NO \| 0.67 \| 0.51, 0.88 \| 0.004 \| \| Spironolactone \|  \|  \|  \| \| YES \| 1.00 \| Reference \|  \| \| NO \| 0.69 \| 0.38, 1.27 \| 0.23 \| \| Statin \|  \|  \|  \| \| YES \| 1.00 \| Reference \|  \| \| NO \| 1.64 \| 1.25, 2.17 \| <0.001 \| \| Vasopressor \|  \|  \|  \| \| YES \| 1.00 \| Reference \|  \| \| NO \| 0.65 \| 0.51, 0.84 \| <0.001 \| \| HF \|  \|  \|  \| \| YES \| 1.00 \| Reference \|  \| \| NO \| 1.00 \| 0.79, 1.27 \| 0.983 \| \| AFIB \|  \|  \|  \| \| YES \| 1.00 \| Reference \|  \| \| NO \| 0.95 \| 0.70, 1.27 \| 0.716 \| \| Diabetes \|  \|  \|  \| \| YES \| 1.00 \| Reference \|  \| \| NO \| 0.97 \| 0.79, 1.19 \| 0.769 \| \| Renal_Disease \|  \|  \|  \| \| YES \| 1.00 \| Reference \|  \| \| NO \| 1.17 \| 0.90, 1.51 \| 0.233 \| \| Liver_Disease \|  \|  \|  \| \| YES \| 1.00 \| Reference \|  \| \| NO \| 0.43 \| 0.22, 0.84 \| 0.013 \| \| COPD \|  \|  \|  \| \| YES \| 1.00 \| Reference \|  \| \| NO \| 0.66 \| 0.51, 0.84 \| <0.001 \| \| Stroke \|  \|  \|  \| \| YES \| 1.00 \| Reference \|  \| \| NO \| 0.39 \| 0.29, 0.52 \| <0.001 \| \| Malignancy \|  \|  \|  \| \| YES \| 1.00 \| Reference \|  \| \| NO \| 0.58 \| 0.43, 0.78 \| <0.001 \| \| MAP \| 1.00 \| 0.99, 1.00 \| 0.428 \| \| Heart_Rate \| 1.01 \| 1.01, 1.02 \| <0.001 \| \| Temperature \| 0.82 \| 0.72, 0.94 \| 0.004 \| \| WBC \| 1.00 \| 0.99, 1.01 \| 0.782 \| \| Hemoglobin \| 0.99 \| 0.94, 1.04 \| 0.684 \| \| Platelet \| 1.00 \| 1.00, 1.00 \| <0.001 \| \| Sodium \| 1.09 \| 1.06, 1.12 \| <0.001 \| \| Potassium \| 0.92 \| 0.81, 1.05 \| 0.208 \| \| Bicarbonate \| 0.93 \| 0.90, 0.95 \| <0.001 \| \| Chloride \| 0.91 \| 0.89, 0.94 \| <0.001 \| \| BUN \| 1.00 \| 1.00, 1.01 \| 0.103 \| \| eGFR \| 1.00 \| 1.0, 1.00 \| 0.911 \| \| BNP_tag \|  \|  \|  \| \| YES \| 1.00 \| Reference \|  \| \| NO \| 1.35 \| 0.91, 1.99 \| 0.133 \| \| TNT_tag \|  \|  \|  \| \| YES \| 1.00 \| Reference \|  \| \| NO \| 0.98 \| 0.75, 1.28 \| 0.878 \| \| CK_tag \|  \|  \|  \| \| YES \| 1.00 \| Reference \|  \| \| NO \| 0.74 \| 0.59, 0.93 \| 0.01 \| \| ^1^OR = Odds Ratio, CI = Confidence Interval \| \| \| \| | | | |

Supplementary Table 11. Survey-weighted generalised linear model adjusted with unbalanced covariates and IPTW for 28-day mortality of the Non-AKI and Mild-AKI cohorts

| **Characteristic** | **OR**^1^ | **95% CI**^1^ | **p-value** |
| --- | --- | --- | --- |
| Group |  |  |  |
| Non-AKI | 1.00 | Reference |  |
| Mild-AKI | 0.97 | 0.68, 1.38 | 0.854 |
| Age | 1.05 | 1.04, 1.07 | <0.001 |
| SOFA_Score | 1.32 | 1.22, 1.43 | <0.001 |
| Coronary_Artery_Bypass_Grafting |  |  |  |
| YES | 1.00 | Reference |  |
| NO | 7.82 | 1.77, 34.6 | 0.007 |
| Percutaneous_Coronary_Intervention |  |  |  |
| YES | 1.00 | Reference |  |
| NO | 1.11 | 0.59, 2.07 | 0.749 |
| Continuous_Renal_Replacement_Therapy |  |  |  |
| YES | 1.00 | Reference |  |
| NO | 0.87 | 0.24, 3.10 | 0.824 |
| IABP |  |  |  |
| YES | 1.00 | Reference |  |
| NO | 0.69 | 0.41, 1.16 | 0.164 |
| B_blocker |  |  |  |
| YES | 1.00 | Reference |  |
| NO | 2.46 | 1.72, 3.52 | <0.001 |
| Loop_diuretic |  |  |  |
| YES | 1.00 | Reference |  |
| NO | 0.93 | 0.61, 1.41 | 0.722 |
| Positive_inotropic |  |  |  |
| YES | 1.00 | Reference |  |
| NO | 0.99 | 0.56, 1.76 | 0.979 |
| Statin |  |  |  |
| YES | 1.00 | Reference |  |
| NO | 2.44 | 1.68, 3.53 | <0.001 |
| Vasopressor |  |  |  |
| YES | 1.00 | Reference |  |
| NO | 0.90 | 0.55, 1.45 | 0.652 |
| HF |  |  |  |
| YES | 1.00 | Reference |  |
| NO | 1.09 | 0.74, 1.60 | 0.677 |
| Diabetes |  |  |  |
| YES | 1.00 | Reference |  |
| NO | 1.05 | 0.73, 1.52 | 0.788 |
| Renal |  |  |  |
| YES | 1.00 | Reference |  |
| NO | 1.08 | 0.68, 1.71 | 0.743 |
| MAP | 1.00 | 0.99, 1.01 | 0.386 |
| Hemoglobin | 1.05 | 0.97, 1.14 | 0.2 |
| Platelet | 1.00 | 1.00, 1.00 | 0.005 |
| Sodium | 1.01 | 0.97, 1.04 | 0.642 |
| Potassium | 0.93 | 0.75, 1.15 | 0.492 |
| Bicarbonate | 0.93 | 0.88, 0.97 | <0.001 |
| BUN | 1.00 | 0.99, 1.01 | 0.695 |
| eGFR | 1.00 | 0.99, 1.00 | 0.316 |
| TNT_tag |  |  |  |
| YES | 1.00 | Reference |  |
| NO | 0.99 | 0.65, 1.52 | 0.963 |
| CK_tag |  |  |  |
| YES | 1.00 | Reference |  |
| NO | 0.89 | 0.59, 1.35 | 0.587 |
| ^1^OR = Odds Ratio, CI = Confidence Interval  Supplementary Table 12. Survey-weighted generalised linear model adjusted with unbalanced covariates and IPTW for 28-day mortality of the Normal-or-mild-AKI and Moderate-to-severe-AKI cohorts   \| **Characteristic** \| **OR**^1^ \| **95% CI**^1^ \| **p-value** \| \| --- \| --- \| --- \| --- \| \| Group \|  \|  \|  \| \| Normal-or-mild-AKI \| 1.00 \| Reference \|  \| \| Moderate-to-severe-AKI \| 1.67 \| 1.36, 2.05 \| <0.001 \| \| Age \| 1.05 \| 1.05, 1.06 \| <0.001 \| \| SOFA_Score \| 1.21 \| 1.17, 1.25 \| <0.001 \| \| Percutaneous_Coronary_Intervention \|  \|  \|  \| \| YES \| 1.00 \| Reference \|  \| \| NO \| 0.76 \| 0.54, 1.06 \| 0.105 \| \| Continuous_Renal_Replacement_Therapy \|  \|  \|  \| \| YES \| 1.00 \| Reference \|  \| \| NO \| 0.84 \| 0.51, 1.37 \| 0.483 \| \| IABP \|  \|  \|  \| \| YES \| 1.00 \| Reference \|  \| \| NO \| 0.80 \| 0.59, 1.08 \| 0.147 \| \| Anticoagulant \|  \|  \|  \| \| YES \| 1.00 \| Reference \|  \| \| NO \| 0.83 \| 0.63, 1.09 \| 0.184 \| \| Loop_diuretic \|  \|  \|  \| \| YES \| 1.00 \| Reference \|  \| \| NO \| 1.24 \| 1.00, 1.54 \| 0.051 \| \| Statin \|  \|  \|  \| \| YES \| 1.00 \| Reference \|  \| \| NO \| 2.77 \| 2.19, 3.50 \| <0.001 \| \| Vasopressor \|  \|  \|  \| \| YES \| 1.00 \| Reference \|  \| \| NO \| 0.69 \| 0.54, 0.88 \| 0.003 \| \| HF \|  \|  \|  \| \| YES \| 1.00 \| Reference \|  \| \| NO \| 0.94 \| 0.76, 1.15 \| 0.537 \| \| AFIB \|  \|  \|  \| \| YES \| 1.00 \| Reference \|  \| \| NO \| 1.05 \| 0.79, 1.38 \| 0.757 \| \| Diabetes \|  \|  \|  \| \| YES \| 1.00 \| Reference \|  \| \| NO \| 1.07 \| 0.88, 1.30 \| 0.519 \| \| Renal_Disease \|  \|  \|  \| \| YES \| 1.00 \| Reference \|  \| \| NO \| 1.35 \| 1.06, 1.73 \| 0.015 \| \| Stroke \|  \|  \|  \| \| YES \| 1.00 \| Reference \|  \| \| NO \| 0.44 \| 0.33, 0.58 \| <0.001 \| \| Heart_Rate \| 1.01 \| 1.01, 1.02 \| <0.001 \| \| WBC \| 1.01 \| 1.00, 1.02 \| 0.07 \| \| Bicarbonate \| 0.94 \| 0.92, 0.96 \| <0.001 \| \| Chloride \| 0.95 \| 0.94, 0.97 \| <0.001 \| \| BUN \| 1.01 \| 1.00, 1.01 \| <0.001 \| \| eGFR \| 1.00 \| 0.99, 1.00 \| 0.07 \| \| TNT_tag \|  \|  \|  \| \| YES \| 1.00 \| Reference \|  \| \| NO \| 0.64 \| 0.49, 0.83 \| 0.001 \| \| CK_tag \|  \|  \|  \| \| YES \| 1.00 \| Reference \|  \| \| NO \| 0.77 \| 0.62, 0.97 \| 0.028 \| \| ^1^OR = Odds Ratio, CI = Confidence Interval \| \| \| \| | | | |

Supplementary Table 13. Multivariate Cox model adjusted with all covariates for 180-day mortality of the Non-AKI and Mild-AKI cohorts

| **Characteristic** | **HR^1^** | **95% CI^1^** | **p-value** |
| --- | --- | --- | --- |
| Group |  |  |  |
| Non-AKI | 1.00 | Reference |  |
| Mild-AKI | 0.95 | 0.77, 1.18 | 0.671 |
| Age (time dependent) | 1.01 | 1.01, 1.02 | <0.001 |
| Gender |  |  |  |
| Female | 1.00 | Reference |  |
| Male | 1.18 | 0.95, 1.46 | 0.135 |
| SOFA_Score (time dependent) | 1.04 | 1.03, 1.06 | <0.001 |
| Coronary_Artery_Bypass_Grafting |  |  |  |
| YES | 1.00 | Reference |  |
| NO | 12.31 | 4.43, 34.23 | <0.001 |
| Percutaneous_Coronary_Intervention |  |  |  |
| YES | 1.00 | Reference |  |
| NO | 1.08 | 0.73, 1.61 | 0.701 |
| Continuous_Renal_Replacement_Therapy |  |  |  |
| YES | 1.00 | Reference |  |
| NO | 1.01 | 0.47, 2.19 | 0.975 |
| IABP |  |  |  |
| YES | 1.00 | Reference |  |
| NO | 0.50 | 0.32, 0.76 | 0.001 |
| ACEIARB (time dependent) |  |  |  |
| YES | 1.00 | Reference |  |
| NO | 1.07 | 0.99, 1.15 | 0.103 |
| Anticoagulant (time dependent) |  |  |  |
| YES | 1.00 | Reference |  |
| NO | 0.93 | 0.86, 1.01 | 0.082 |
| Antiplatelet (time dependent) |  |  |  |
| YES | 1.00 | Reference |  |
| NO | 1.11 | 1.04, 1.19 | 0.003 |
| B_blocker (time dependent) |  |  |  |
| YES | 1.00 | Reference |  |
| NO | 1.13 | 1.06, 1.20 | <0.001 |
| Loop_diuretic (time dependent) |  |  |  |
| YES | 1.00 | Reference |  |
| NO | 0.97 | 0.91, 1.03 | 0.263 |
| Positive_inotropic |  |  |  |
| YES | 1.00 | Reference |  |
| NO | 1.37 | 0.92, 2.06 | 0.124 |
| Spironolactone |  |  |  |
| YES | 1.00 | Reference |  |
| NO | 0.91 | 0.45, 1.81 | 0.785 |
| Statin (time dependent) |  |  |  |
| YES | 1.00 | Reference |  |
| NO | 1.07 | 1.00, 1.14 | 0.051 |
| Vasopressor (time dependent) |  |  |  |
| YES | 1.00 | Reference |  |
| NO | 1.06 | 0.99, 1.14 | 0.106 |
| HF (time dependent) |  |  |  |
| YES | 1.00 | Reference |  |
| NO | 1.01 | 0.95, 1.07 | 0.743 |
| AFIB |  |  |  |
| YES | 1.00 | Reference |  |
| NO | 1.26 | 0.88, 1.80 | 0.205 |
| Diabetes (time dependent) |  |  |  |
| YES | 1.00 | Reference |  |
| NO | 0.99 | 0.93, 1.05 | 0.68 |
| Renal (time dependent) |  |  |  |
| YES | 1.00 | Reference |  |
| NO | 0.99 | 0.92, 1.06 | 0.734 |
| Liver |  |  |  |
| YES | 1.00 | Reference |  |
| NO | 0.65 | 0.28, 1.49 | 0.311 |
| COPD |  |  |  |
| YES | 1.00 | Reference |  |
| NO | 0.97 | 0.74, 1.29 | 0.853 |
| Stroke |  |  |  |
| YES | 1.00 | Reference |  |
| NO | 0.36 | 0.27, 0.49 | <0.001 |
| Malignancy (time dependent) |  |  |  |
| YES | 1.00 | Reference |  |
| NO | 0.90 | 0.85, 0.96 | 0.002 |
| MAP (time dependent) | 1.00 | 1.00, 1.00 | 0.004 |
| Heart_Rate | 1.01 | 1.01, 1.02 | <0.001 |
| Temperature (time dependent) | 0.91 | 0.89, 0.94 | <0.001 |
| WBC | 1.00 | 0.99, 1.01 | 0.995 |
| Hemoglobin (time dependent) | 0.99 | 0.98, 1.00 | 0.143 |
| Platelet (time dependent) | 1.00 | 1.00, 1.00 | 0.002 |
| Sodium | 1.07 | 1.04, 1.10 | <0.001 |
| Potassium | 1.07 | 0.93, 1.23 | 0.35 |
| Bicarbonate (time dependent) | 0.99 | 0.98, 0.99 | <0.001 |
| Chloride | 0.95 | 0.93, 0.97 | <0.001 |
| BUN | 1.00 | 0.99, 1.00 | 0.32 |
| eGFR | 1.00 | 0.99, 1.00 | 0.191 |
| BNP_tag |  |  |  |
| YES | 1.00 | Reference |  |
| NO | 0.86 | 0.56, 1.32 | 0.491 |
| TNT_tag (time dependent) |  |  |  |
| YES | 1.00 | Reference |  |
| NO | 1.00 | 0.93, 1.07 | 0.933 |
| CK_tag |  |  |  |
| YES | 1.00 | Reference |  |
| NO | 0.96 | 0.76, 1.22 | 0.754 |
| ^1^HR = Hazard Ratio, CI = Confidence Interval  Supplementary Table 14. Multivariate Cox model adjusted with all covariates for 180-day mortality of the Normal-or-mild-AKI and Moderate-to-severe-AKI cohorts   \| **Characteristic** \| **HR^1^** \| **95% CI^1^** \| **p-value** \| \| --- \| --- \| --- \| --- \| \| Group (time dependent) \|  \|  \|  \| \| Normal-or-mild-AKI \| 1.00 \| Reference \|  \| \| Moderate-to-severe-AKI \| 1.09 \| 1.06, 1.13 \| <0.001 \| \| Age (time dependent) \| 1.01 \| 1.01, 1.01 \| <0.001 \| \| Gender \|  \|  \|  \| \| Female \| 1.00 \| Reference \|  \| \| Male \| 0.96 \| 0.85, 1.07 \| 0.446 \| \| SOFA_Score (time dependent) \| 1.03 \| 1.02, 1.03 \| <0.001 \| \| Coronary_Artery_Bypass_Grafting (time dependent) \|  \|  \|  \| \| YES \| 1.00 \| Reference \|  \| \| NO \| 1.42 \| 1.30, 1.55 \| <0.001 \| \| Percutaneous_Coronary_Intervention (time dependent) \|  \|  \|  \| \| YES \| 1.00 \| Reference \|  \| \| NO \| 1.01 \| 0.95, 1.07 \| 0.766 \| \| Continuous_Renal_Replacement_Therapy \|  \|  \|  \| \| YES \| 1.00 \| Reference \|  \| \| NO \| 0.85 \| 0.66, 1.10 \| 0.224 \| \| IABP (time dependent) \|  \|  \|  \| \| YES \| 1.00 \| Reference \|  \| \| NO \| 0.97 \| 0.92, 1.02 \| 0.196 \| \| ACEIARB (time dependent) \|  \|  \|  \| \| YES \| 1.00 \| Reference \|  \| \| NO \| 1.11 \| 1.07, 1.16 \| <0.001 \| \| Anticoagulant (time dependent) \|  \|  \|  \| \| YES \| 1.00 \| Reference \|  \| \| NO \| 0.96 \| 0.91, 1.01 \| 0.142 \| \| Antiplatelet (time dependent) \|  \|  \|  \| \| YES \| 1.00 \| Reference \|  \| \| NO \| 1.02 \| 0.97, 1.06 \| 0.498 \| \| B_blocker (time dependent) \|  \|  \|  \| \| YES \| 1.00 \| Reference \|  \| \| NO \| 1.19 \| 1.15, 1.23 \| <0.001 \| \| Loop_diuretic (time dependent) \|  \|  \|  \| \| YES \| 1.00 \| Reference \|  \| \| NO \| 1.00 \| 0.97, 1.04 \| 0.897 \| \| Positive_inotropic (time dependent) \|  \|  \|  \| \| YES \| 1.00 \| Reference \|  \| \| NO \| 0.96 \| 0.92, 1.00 \| 0.059 \| \| Spironolactone (time dependent) \|  \|  \|  \| \| YES \| 1.00 \| Reference \|  \| \| NO \| 0.85 \| 0.79, 0.93 \| <0.001 \| \| Statin (time dependent) \|  \|  \|  \| \| YES \| 1.00 \| Reference \|  \| \| NO \| 1.05 \| 1.01, 1.09 \| 0.019 \| \| Vasopressor (time dependent) \|  \|  \|  \| \| YES \| 1.00 \| Reference \|  \| \| NO \| 0.96 \| 0.92, 0.99 \| 0.017 \| \| HF (time dependent) \|  \|  \|  \| \| YES \| 1.00 \| Reference \|  \| \| NO \| 0.95 \| 0.92, 0.98 \| 0.003 \| \| AFIB (time dependent) \|  \|  \|  \| \| YES \| 1.00 \| Reference \|  \| \| NO \| 0.98 \| 0.94, 1.03 \| 0.428 \| \| Diabetes (time dependent) \|  \|  \|  \| \| YES \| 1.00 \| Reference \|  \| \| NO \| 0.99 \| 0.96, 1.02 \| 0.367 \| \| Renal_Disease (time dependent) \|  \|  \|  \| \| YES \| 1.00 \| Reference \|  \| \| NO \| 1.03 \| 0.99, 1.07 \| 0.13 \| \| Liver_Disease \|  \|  \|  \| \| YES \| 1.00 \| Reference \|  \| \| NO \| 0.64 \| 0.45, 0.92 \| 0.016 \| \| COPD \|  \|  \|  \| \| YES \| 1.00 \| Reference \|  \| \| NO \| 0.76 \| 0.66, 0.87 \| <0.001 \| \| Stroke (time dependent) \|  \|  \|  \| \| YES \| 1.00 \| Reference \|  \| \| NO \| 0.86 \| 0.83, 0.89 \| <0.001 \| \| Malignancy (time dependent) \|  \|  \|  \| \| YES \| 1.00 \| Reference \|  \| \| NO \| 0.92 \| 0.89, 0.95 \| <0.001 \| \| MAP (time dependent) \| 1.00 \| 1.00, 1.00 \| 0.57 \| \| Heart_Rate \| 1.01 \| 1.01, 1.01 \| <0.001 \| \| Temperature (time dependent) \| 0.96 \| 0.94, 0.97 \| <0.001 \| \| WBC (time dependent) \| 1.00 \| 1.00, 1.00 \| 0.974 \| \| Hemoglobin (time dependent) \| 0.99 \| 0.99, 1.00 \| 0.085 \| \| Platelet \| 1.00 \| 1.00, 1.00 \| <0.001 \| \| Sodium \| 1.04 \| 1.03, 1.05 \| <0.001 \| \| Potassium \| 1.02 \| 0.95, 1.10 \| 0.64 \| \| Bicarbonate (time dependent) \| 0.99 \| 0.99, 1.00 \| <0.001 \| \| Chloride \| 0.96 \| 0.95, 0.97 \| <0.001 \| \| BUN \| 1.00 \| 1.00, 1.01 \| 0.005 \| \| eGFR (time dependent) \| 1.00 \| 1.00, 1.00 \| 0.01 \| \| BNP_tag \|  \|  \|  \| \| YES \| 1.00 \| Reference \|  \| \| NO \| 1.04 \| 0.83, 1.30 \| 0.753 \| \| TNT_tag \|  \|  \|  \| \| YES \| 1.00 \| Reference \|  \| \| NO \| 0.97 \| 0.83, 1.13 \| 0.668 \| \| CK_tag \|  \|  \|  \| \| YES \| 1.00 \| Reference \|  \| \| NO \| 0.84 \| 0.74, 0.96 \| 0.009 \| \| ^1^HR = Hazard Ratio, CI = Confidence Interval \| \| \| \| | | | |

Supplementary Table 15. Multivariate Cox model adjusted with unbalanced covariates and IPTW for 180-day mortality of the Non-AKI and Mild-AKI cohorts

| **Characteristic** | **HR^1^** | **95% CI^1^** | **p-value** |
| --- | --- | --- | --- |
| Group |  |  |  |
| Non-AKI | 1.00 | Reference |  |
| Mild-AKI | 1.02 | 0.82, 1.27 | 0.839 |
| Age (time dependent) | 1.01 | 1.01, 1.02 | <0.001 |
| SOFA_Score (time dependent) | 1.05 | 1.04, 1.06 | <0.001 |
| Coronary_Artery_Bypass_Grafting |  |  |  |
| YES | 1.00 | Reference |  |
| NO | 12.65 | 4.60, 34.81 | <0.001 |
| Percutaneous_Coronary_Intervention |  |  |  |
| YES | 1.00 | Reference |  |
| NO | 1.32 | 0.90, 1.95 | 0.16 |
| Continuous_Renal_Replacement_Therapy |  |  |  |
| YES | 1.00 | Reference |  |
| NO | 0.66 | 0.33, 1.34 | 0.255 |
| IABP |  |  |  |
| YES | 1.00 | Reference |  |
| NO | 0.56 | 0.37, 0.86 | 0.007 |
| B_blocker (time dependent) |  |  |  |
| YES | 1.00 | Reference |  |
| NO | 1.15 | 1.08, 1.22 | <0.001 |
| Loop_diuretic (time dependent) |  |  |  |
| YES | 1.00 | Reference |  |
| NO | 0.99 | 0.94, 1.06 | 0.846 |
| Positive_inotropic |  |  |  |
| YES | 1.00 | Reference |  |
| NO | 1.60 | 1.07, 2.38 | 0.021 |
| Statin (time dependent) |  |  |  |
| YES | 1.00 | Reference |  |
| NO | 1.11 | 1.04, 1.18 | 0.001 |
| Vasopressor (time dependent) |  |  |  |
| YES | 1.00 | Reference |  |
| NO | 1.07 | 1.00, 1.15 | 0.051 |
| HF (time dependent) |  |  |  |
| YES | 1.00 | Reference |  |
| NO | 0.98 | 0.93, 1.04 | 0.581 |
| Diabetes (time dependent) |  |  |  |
| YES | 1.00 | Reference |  |
| NO | 0.98 | 0.92, 1.03 | 0.384 |
| Renal (time dependent) |  |  |  |
| YES | 1.00 | Reference |  |
| NO | 0.99 | 0.93, 1.05 | 0.702 |
| MAP (time dependent) | 1.00 | 1.00, 1.00 | 0.107 |
| Hemoglobin (time dependent) | 1.00 | 0.98, 1.01 | 0.667 |
| Platelet | 1.00 | 1.00, 1.00 | <0.001 |
| Sodium | 1.02 | 1.00, 1.04 | 0.054 |
| Potassium | 1.05 | 0.91, 1.21 | 0.533 |
| Bicarbonate (time dependent) | 0.99 | 0.98, 0.99 | <0.001 |
| BUN | 1.00 | 0.99, 1.00 | 0.869 |
| eGFR | 1.00 | 0.99, 1.00 | 0.269 |
| TNT_tag (time dependent) |  |  |  |
| YES | 1.00 | Reference |  |
| NO | 0.97 | 0.91, 1.04 | 0.417 |
| CK_tag |  |  |  |
| YES | 1.00 | Reference |  |
| NO | 0.96 | 0.77, 1.20 | 0.722 |
| ^1^HR = Hazard Ratio, CI = Confidence Interval  Supplementary Table 16. Multivariate Cox model adjusted with unbalanced covariates and IPTW for 180-day mortality of the Normal-or-mild-AKI and Moderate-to-severe-AKI cohorts   \| **Characteristic** \| **HR^1^** \| **95% CI^1^** \| **p-value** \| \| --- \| --- \| --- \| --- \| \| Group (time dependent) \|  \|  \|  \| \| Normal-or-mild-AKI \| 1.00 \| Reference \|  \| \| Moderate-to-severe-AKI \| 1.07 \| 1.03, 1.10 \| <0.001 \| \| Age (time dependent) \| 1.01 \| 1.01, 1.01 \| <0.001 \| \| SOFA_Score (time dependent) \| 1.03 \| 1.02, 1.03 \| <0.001 \| \| Percutaneous_Coronary_Intervention (time dependent) \|  \|  \|  \| \| YES \| 1.00 \| Reference \|  \| \| NO \| 1.02 \| 0.96, 1.08 \| 0.571 \| \| Continuous_Renal_Replacement_Therapy \|  \|  \|  \| \| YES \| 1.00 \| Reference \|  \| \| NO \| 0.82 \| 0.63, 1.05 \| 0.118 \| \| IABP (time dependent) \|  \|  \|  \| \| YES \| 1.00 \| Reference \|  \| \| NO \| 0.99 \| 0.95, 1.04 \| 0.81 \| \| Anticoagulant (time dependent) \|  \|  \|  \| \| YES \| 1.00 \| Reference \|  \| \| NO \| 0.95 \| 0.90, 1.00 \| 0.075 \| \| Loop_diuretic (time dependent) \|  \|  \|  \| \| YES \| 1.00 \| Reference \|  \| \| NO \| 1.04 \| 1.01, 1.08 \| 0.017 \| \| Statin (time dependent) \|  \|  \|  \| \| YES \| 1.00 \| Reference \|  \| \| NO \| 1.14 \| 1.10, 1.18 \| <0.001 \| \| Vasopressor (time dependent) \|  \|  \|  \| \| YES \| 1.00 \| Reference \|  \| \| NO \| 0.97 \| 0.94, 1.01 \| 0.177 \| \| HF (time dependent) \|  \|  \|  \| \| YES \| 1.00 \| Reference \|  \| \| NO \| 0.93 \| 0.90, 0.97 \| <0.001 \| \| AFIB (time dependent) \|  \|  \|  \| \| YES \| 1.00 \| Reference \|  \| \| NO \| 0.97 \| 0.93, 1.01 \| 0.149 \| \| Diabetes (time dependent) \|  \|  \|  \| \| YES \| 1.00 \| Reference \|  \| \| NO \| 0.99 \| 0.96, 1.02 \| 0.715 \| \| Renal_Disease (time dependent) \|  \|  \|  \| \| YES \| 1.00 \| Reference \|  \| \| NO \| 1.04 \| 1.01, 1.08 \| 0.019 \| \| Stroke (time dependent) \|  \|  \|  \| \| YES \| 1.00 \| Reference \|  \| \| NO \| 0.87 \| 0.84, 0.90 \| <0.001 \| \| Heart_Rate (time dependent) \| 1.00 \| 1.00, 1.00 \| <0.001 \| \| WBC (time dependent) \| 1.00 \| 1.00, 1.00 \| 0.347 \| \| Bicarbonate (time dependent) \| 0.99 \| 0.99, 1.00 \| <0.001 \| \| Chloride \| 0.97 \| 0.96, 0.98 \| <0.001 \| \| BUN \| 1.01 \| 1.00, 1.01 \| <0.001 \| \| eGFR (time dependent) \| 1.00 \| 1.00, 1.00 \| 0.002 \| \| TNT_tag (time dependent) \|  \|  \|  \| \| YES \| 1.00 \| Reference \|  \| \| NO \| 0.91 \| 0.87, 0.95 \| <0.001 \| \| CK_tag \|  \|  \|  \| \| YES \| 1.00 \| Reference \|  \| \| NO \| 0.88 \| 0.78, 1.00 \| 0.056 \| \| ^1^HR = Hazard Ratio, CI = Confidence Interval \| \| \| \| | | | |

Supplementary Table 17. Survey-weighted Cox model adjusted with all covariates and IPTW for 180-day mortality of the Non-AKI and Mild-AKI cohorts

| **Characteristic** | **HR^1^** | **95% CI^1^** | **p-value** |
| --- | --- | --- | --- |
| Group |  |  |  |
| Non-AKI | 1.00 | Reference |  |
| Mild-AKI | 0.89 | 0.71, 1.11 | 0.309 |
| Age (time dependent) | 1.01 | 1.01, 1.02 | <0.001 |
| Gender |  |  |  |
| Female | 1.00 | Reference |  |
| Male | 1.12 | 0.88, 1.41 | 0.35 |
| SOFA_Score (time dependent) | 1.05 | 1.03, 1.06 | <0.001 |
| Coronary_Artery_Bypass_Grafting |  |  |  |
| YES | 1.00 | Reference |  |
| NO | 7.58 | 2.61, 22.01 | <0.001 |
| Percutaneous_Coronary_Intervention |  |  |  |
| YES | 1.00 | Reference |  |
| NO | 1.07 | 0.70, 1.63 | 0.76 |
| Continuous_Renal_Replacement_Therapy |  |  |  |
| YES | 1.00 | Reference |  |
| NO | 1.05 | 0.42, 2.65 | 0.912 |
| IABP (time dependent) |  |  |  |
| YES | 1.00 | Reference |  |
| NO | 0.85 | 0.75, 0.97 | 0.015 |
| ACEIARB (time dependent) |  |  |  |
| YES | 1.00 | Reference |  |
| NO | 1.05 | 0.97, 1.14 | 0.23 |
| Anticoagulant (time dependent) |  |  |  |
| YES | 1.00 | Reference |  |
| NO | 0.92 | 0.84, 1.00 | 0.061 |
| Antiplatelet (time dependent) |  |  |  |
| YES | 1.00 | Reference |  |
| NO | 1.12 | 1.03, 1.21 | 0.006 |
| B_blocker (time dependent) |  |  |  |
| YES | 1.00 | Reference |  |
| NO | 1.12 | 1.04, 1.20 | 0.002 |
| Loop_diuretic (time dependent) |  |  |  |
| YES | 1.00 | Reference |  |
| NO | 0.95 | 0.88, 1.01 | 0.107 |
| Positive_inotropic |  |  |  |
| YES | 1.00 | Reference |  |
| NO | 1.36 | 0.87, 2.13 | 0.178 |
| Spironolactone |  |  |  |
| YES | 1.00 | Reference |  |
| NO | 0.78 | 0.38, 1.61 | 0.503 |
| Statin (time dependent) |  |  |  |
| YES | 1.00 | Reference |  |
| NO | 1.07 | 0.99, 1.15 | 0.076 |
| Vasopressor (time dependent) |  |  |  |
| YES | 1.00 | Reference |  |
| NO | 1.07 | 0.98, 1.16 | 0.135 |
| HF (time dependent) |  |  |  |
| YES | 1.00 | Reference |  |
| NO | 1.00 | 0.94, 1.06 | 0.945 |
| AFIB |  |  |  |
| YES | 1.00 | Reference |  |
| NO | 1.32 | 0.90, 1.95 | 0.16 |
| Diabetes (time dependent) |  |  |  |
| YES | 1.00 | Reference |  |
| NO | 0.99 | 0.93, 1.05 | 0.723 |
| Renal (time dependent) |  |  |  |
| YES | 1.00 | Reference |  |
| NO | 0.99 | 0.92, 1.07 | 0.847 |
| Liver |  |  |  |
| YES | 1.00 | Reference |  |
| NO | 0.66 | 0.28, 1.59 | 0.359 |
| COPD |  |  |  |
| YES | 1.00 | Reference |  |
| NO | 0.95 | 0.70, 1.29 | 0.755 |
| Stroke |  |  |  |
| YES | 1.00 | Reference |  |
| NO | 0.36 | 0.26, 0.51 | <0.001 |
| Malignancy (time dependent) |  |  |  |
| YES | 1.00 | Reference |  |
| NO | 0.90 | 0.83, 0.97 | 0.009 |
| MAP (time dependent) | 1.00 | 1.00, 1.00 | 0.076 |
| Heart_Rate | 1.01 | 1.00, 1.02 | <0.001 |
| Temperature (time dependent) | 0.91 | 0.88, 0.95 | <0.001 |
| WBC | 1.00 | 1.00, 1.01 | 0.624 |
| Hemoglobin (time dependent) | 0.99 | 0.98, 1.01 | 0.332 |
| Platelet (time dependent) | 1.00 | 1.00, 1.00 | 0.004 |
| Sodium | 1.07 | 1.03, 1.11 | <0.001 |
| Potassium | 1.04 | 0.88, 1.23 | 0.61 |
| Bicarbonate (time dependent) | 0.99 | 0.98, 1.00 | 0.002 |
| Chloride | 0.96 | 0.94, 0.98 | <0.001 |
| BUN | 1.00 | 0.99, 1.00 | 0.26 |
| eGFR | 1.00 | 0.99, 1.00 | 0.218 |
| BNP_tag |  |  |  |
| YES | 1.00 | Reference |  |
| NO | 0.89 | 0.57, 1.39 | 0.601 |
| TNT_tag (time dependent) |  |  |  |
| YES | 1.00 | Reference |  |
| NO | 0.99 | 0.91, 1.08 | 0.875 |
| CK_tag |  |  |  |
| YES | 1.00 | Reference |  |
| NO | 0.98 | 0.76, 1.27 | 0.902 |
| ^1^HR = Hazard Ratio, CI = Confidence Interval  Supplementary Table 18. Multivariate Cox model adjusted with all covariates and IPTW for 180-day mortality of the Normal-or-mild-AKI and Moderate-to-severe-AKI cohorts   \| **Characteristic** \| **HR^1^** \| **95% CI^1^** \| **p-value** \| \| --- \| --- \| --- \| --- \| \| Group (time dependent) \|  \|  \|  \| \| Normal-or-mild-AKI \| 1.00 \| Reference \|  \| \| Moderate-to-severe-AKI \| 1.07 \| 1.03, 1.11 \| <0.001 \| \| Age (time dependent) \| 1.01 \| 1.01, 1.01 \| <0.001 \| \| Gender (time dependent) \|  \|  \|  \| \| Female \| 1.00 \| Reference \|  \| \| Male \| 0.99 \| 0.96, 1.02 \| 0.558 \| \| SOFA_Score (time dependent) \| 1.03 \| 1.03, 1.04 \| <0.001 \| \| Coronary_Artery_Bypass_Grafting (time dependent) \|  \|  \|  \| \| YES \| 1.00 \| Reference \|  \| \| NO \| 1.45 \| 1.30, 1.62 \| <0.001 \| \| Percutaneous_Coronary_Intervention (time dependent) \|  \|  \|  \| \| YES \| 1.00 \| Reference \|  \| \| NO \| 1.00 \| 0.94, 1.07 \| 0.993 \| \| Continuous_Renal_Replacement_Therapy \|  \|  \|  \| \| YES \| 1.00 \| Reference \|  \| \| NO \| 0.80 \| 0.58, 1.10 \| 0.167 \| \| IABP (time dependent) \|  \|  \|  \| \| YES \| 1.00 \| Reference \|  \| \| NO \| 0.95 \| 0.90, 1.01 \| 0.131 \| \| ACEIARB (time dependent) \|  \|  \|  \| \| YES \| 1.00 \| Reference \|  \| \| NO \| 1.10 \| 1.05, 1.15 \| <0.001 \| \| Anticoagulant (time dependent) \|  \|  \|  \| \| YES \| 1.00 \| Reference \|  \| \| NO \| 0.97 \| 0.91, 1.03 \| 0.303 \| \| Antiplatelet (time dependent) \|  \|  \|  \| \| YES \| 1.00 \| Reference \|  \| \| NO \| 1.02 \| 0.98, 1.07 \| 0.325 \| \| B_blocker (time dependent) \|  \|  \|  \| \| YES \| 1.00 \| Reference \|  \| \| NO \| 1.17 \| 1.12, 1.21 \| <0.001 \| \| Loop_diuretic (time dependent) \|  \|  \|  \| \| YES \| 1.00 \| Reference \|  \| \| NO \| 1.00 \| 0.96, 1.04 \| 0.961 \| \| Positive_inotropic (time dependent) \|  \|  \|  \| \| YES \| 1.00 \| Reference \|  \| \| NO \| 0.96 \| 0.91, 1.01 \| 0.084 \| \| Spironolactone (time dependent) \|  \|  \|  \| \| YES \| 1.00 \| Reference \|  \| \| NO \| 0.86 \| 0.78, 0.94 \| <0.001 \| \| Statin (time dependent) \|  \|  \|  \| \| YES \| 1.00 \| Reference \|  \| \| NO \| 1.06 \| 1.01, 1.10 \| 0.013 \| \| Vasopressor (time dependent) \|  \|  \|  \| \| YES \| 1.00 \| Reference \|  \| \| NO \| 0.97 \| 0.93, 1.01 \| 0.11 \| \| HF (time dependent) \|  \|  \|  \| \| YES \| 1.00 \| Reference \|  \| \| NO \| 0.96 \| 0.92, 1.00 \| 0.027 \| \| AFIB (time dependent) \|  \|  \|  \| \| YES \| 1.00 \| Reference \|  \| \| NO \| 1.00 \| 0.96, 1.05 \| 0.902 \| \| Diabetes (time dependent) \|  \|  \|  \| \| YES \| 1.00 \| Reference \|  \| \| NO \| 0.98 \| 0.95, 1.02 \| 0.298 \| \| Renal_Disease (time dependent) \|  \|  \|  \| \| YES \| 1.00 \| Reference \|  \| \| NO \| 1.01 \| 0.97, 1.06 \| 0.632 \| \| Liver_Disease \|  \|  \|  \| \| YES \| 1.00 \| Reference \|  \| \| NO \| 0.55 \| 0.37, 0.83 \| 0.004 \| \| COPD \|  \|  \|  \| \| YES \| 1.00 \| Reference \|  \| \| NO \| 0.78 \| 0.66, 0.91 \| 0.002 \| \| Stroke (time dependent) \|  \|  \|  \| \| YES \| 1.00 \| Reference \|  \| \| NO \| 0.84 \| 0.80, 0.88 \| <0.001 \| \| Malignancy (time dependent) \|  \|  \|  \| \| YES \| 1.00 \| Reference \|  \| \| NO \| 0.92 \| 0.88, 0.96 \| <0.001 \| \| MAP (time dependent) \| 1.00 \| 1.00, 1.00 \| 0.392 \| \| Heart_Rate (time dependent) \| 1.00 \| 1.00, 1.00 \| <0.001 \| \| Temperature (time dependent) \| 0.96 \| 0.94, 0.98 \| <0.001 \| \| WBC (time dependent) \| 1.00 \| 1.00, 1.00 \| 0.712 \| \| Hemoglobin (time dependent) \| 0.99 \| 0.99, 1.00 \| 0.103 \| \| Platelet \| 1.00 \| 1.00, 1.00 \| <0.001 \| \| Sodium \| 1.04 \| 1.03, 1.06 \| <0.001 \| \| Potassium \| 1.00 \| 0.91, 1.09 \| 0.921 \| \| Bicarbonate (time dependent) \| 0.99 \| 0.99, 1.00 \| <0.001 \| \| Chloride (time dependent) \| 0.99 \| 0.99, 0.99 \| <0.001 \| \| BUN \| 1.00 \| 1.00, 1.01 \| 0.016 \| \| eGFR (time dependent) \| 1.00 \| 1.00, 1.00 \| 0.256 \| \| BNP_tag (time dependent) \|  \|  \|  \| \| YES \| 1.00 \| Reference \|  \| \| NO \| 1.00 \| 0.93, 1.07 \| 0.982 \| \| TNT_tag (time dependent) \|  \|  \|  \| \| YES \| 1.00 \| Reference \|  \| \| NO \| 0.98 \| 0.93, 1.02 \| 0.311 \| \| CK_tag \|  \|  \|  \| \| YES \| 1.00 \| Reference \|  \| \| NO \| 0.86 \| 0.75, 0.99 \| 0.039 \| \| ^1^HR = Hazard Ratio, CI = Confidence Interval \| \| \| \| | | | |

Supplementary Table 19. Survey-weighted Cox model adjusted with unbalanced covariates and IPTW for 180-day mortality of the Non-AKI and Mild-AKI cohorts

| **Characteristic** | **HR^1^** | **95% CI^1^** | **p-value** |
| --- | --- | --- | --- |
| Group |  |  |  |
| Non-AKI | 1.00 | Reference |  |
| Mild-AKI | 0.94 | 0.76, 1.18 | 0.618 |
| Age (time dependent) | 1.01 | 1.01, 1.02 | <0.001 |
| SOFA_Score (time dependent) | 1.05 | 1.04, 1.07 | <0.001 |
| Coronary_Artery_Bypass_Grafting |  |  |  |
| YES | 1.00 | Reference |  |
| NO | 7.95 | 2.79, 22.66 | <0.001 |
| Percutaneous_Coronary_Intervention |  |  |  |
| YES | 1.00 | Reference |  |
| NO | 1.27 | 0.84, 1.93 | 0.26 |
| Continuous_Renal_Replacement_Therapy |  |  |  |
| YES | 1.00 | Reference |  |
| NO | 0.69 | 0.32, 1.48 | 0.344 |
| IABP (time dependent) |  |  |  |
| YES | 1.00 | Reference |  |
| NO | 0.89 | 0.78, 1.01 | 0.061 |
| B_blocker (time dependent) |  |  |  |
| YES | 1.00 | Reference |  |
| NO | 1.13 | 1.07, 1.21 | <0.001 |
| Loop_diuretic (time dependent) |  |  |  |
| YES | 1.00 | Reference |  |
| NO | 0.98 | 0.92, 1.04 | 0.484 |
| Positive_inotropic |  |  |  |
| YES | 1.00 | Reference |  |
| NO | 1.54 | 1.00, 2.37 | 0.049 |
| Statin (time dependent) |  |  |  |
| YES | 1.00 | Reference |  |
| NO | 1.11 | 1.04, 1.18 | <0.001 |
| Vasopressor (time dependent) |  |  |  |
| YES | 1.00 | Reference |  |
| NO | 1.08 | 0.99, 1.17 | 0.082 |
| HF (time dependent) |  |  |  |
| YES | 1.00 | Reference |  |
| NO | 0.97 | 0.91, 1.02 | 0.255 |
| Diabetes (time dependent) |  |  |  |
| YES | 1.00 | Reference |  |
| NO | 0.97 | 0.92, 1.03 | 0.334 |
| Renal (time dependent) |  |  |  |
| YES | 1.00 | Reference |  |
| NO | 0.99 | 0.92, 1.06 | 0.726 |
| MAP (time dependent) | 1.00 | 1.00, 1.00 | 0.334 |
| Hemoglobin (time dependent) | 1.00 | 0.99, 1.02 | 0.915 |
| Platelet | 1.00 | 1.00, 1.00 | <0.001 |
| Sodium | 1.03 | 1.00, 1.05 | 0.076 |
| Potassium | 1.03 | 0.87, 1.21 | 0.771 |
| Bicarbonate (time dependent) | 0.99 | 0.98, 1.00 | 0.002 |
| BUN | 1.00 | 0.99, 1.01 | 0.686 |
| eGFR | 1.00 | 0.99, 1.00 | 0.378 |
| TNT_tag (time dependent) |  |  |  |
| YES | 1.00 | Reference |  |
| NO | 0.97 | 0.90, 1.05 | 0.451 |
| CK_tag |  |  |  |
| YES | 1.00 | Reference |  |
| NO | 0.96 | 0.76, 1.22 | 0.753 |
| ^1^HR = Hazard Ratio, CI = Confidence Interval  Supplementary Table 20. Survey-weighted Cox model adjusted with unbalanced covariates and IPTW for 180-day mortality of the Normal-or-mild-AKI and Moderate-to-severe-AKI cohorts   \| **Characteristic** \| **HR^1^** \| **95% CI^1^** \| **p-value** \| \| --- \| --- \| --- \| --- \| \| Group (time dependent) \|  \|  \|  \| \| Normal-or-mild-AKI \| 1.00 \| Reference \|  \| \| Moderate-to-severe-AKI \| 1.06 \| 1.02, 1.10 \| 0.002 \| \| Age (time dependent) \| 1.01 \| 1.01, 1.01 \| <0.001 \| \| SOFA_Score (time dependent) \| 1.03 \| 1.03, 1.04 \| <0.001 \| \| Percutaneous_Coronary_Intervention (time dependent) \|  \|  \|  \| \| YES \| 1.00 \| Reference \|  \| \| NO \| 1.01 \| 0.95, 1.07 \| 0.803 \| \| Continuous_Renal_Replacement_Therapy \|  \|  \|  \| \| YES \| 1.00 \| Reference \|  \| \| NO \| 0.79 \| 0.57, 1.08 \| 0.134 \| \| IABP (time dependent) \|  \|  \|  \| \| YES \| 1.00 \| Reference \|  \| \| NO \| 0.98 \| 0.92, 1.03 \| 0.4 \| \| Anticoagulant (time dependent) \|  \|  \|  \| \| YES \| 1.00 \| Reference \|  \| \| NO \| 0.96 \| 0.91, 1.02 \| 0.158 \| \| Loop_diuretic (time dependent) \|  \|  \|  \| \| YES \| 1.00 \| Reference \|  \| \| NO \| 1.04 \| 1.01, 1.08 \| 0.022 \| \| Statin (time dependent) \|  \|  \|  \| \| YES \| 1.00 \| Reference \|  \| \| NO \| 1.14 \| 1.10, 1.18 \| <0.001 \| \| Vasopressor (time dependent) \|  \|  \|  \| \| YES \| 1.00 \| Reference \|  \| \| NO \| 0.99 \| 0.95, 1.03 \| 0.58 \| \| HF (time dependent) \|  \|  \|  \| \| YES \| 1.00 \| Reference \|  \| \| NO \| 0.94 \| 0.91, 0.97 \| <0.001 \| \| AFIB (time dependent) \|  \|  \|  \| \| YES \| 1.00 \| Reference \|  \| \| NO \| 1.00 \| 0.96, 1.05 \| 0.966 \| \| Diabetes (time dependent) \|  \|  \|  \| \| YES \| 1.00 \| Reference \|  \| \| NO \| 0.99 \| 0.96, 1.02 \| 0.586 \| \| Renal_Disease (time dependent) \|  \|  \|  \| \| YES \| 1.00 \| Reference \|  \| \| NO \| 1.03 \| 0.98, 1.07 \| 0.237 \| \| Stroke (time dependent) \|  \|  \|  \| \| YES \| 1.00 \| Reference \|  \| \| NO \| 0.85 \| 0.81, 0.88 \| <0.001 \| \| Heart_Rate (time dependent) \| 1.00 \| 1.00, 1.00 \| <0.001 \| \| WBC (time dependent) \| 1.00 \| 1.00, 1.00 \| 0.242 \| \| Bicarbonate (time dependent) \| 0.99 \| 0.99, 1.00 \| <0.001 \| \| Chloride \| 0.97 \| 0.96, 0.98 \| <0.001 \| \| BUN \| 1.01 \| 1.00, 1.01 \| <0.001 \| \| eGFR (time dependent) \| 1.00 \| 1.00, 1.00 \| 0.081 \| \| TNT_tag (time dependent) \|  \|  \|  \| \| YES \| 1.00 \| Reference \|  \| \| NO \| 0.90 \| 0.85, 0.94 \| <0.001 \| \| CK_tag \|  \|  \|  \| \| YES \| 1.00 \| Reference \|  \| \| NO \| 0.88 \| 0.77, 1.02 \| 0.085 \| \| ^1^HR = Hazard Ratio, CI = Confidence Interval \| \| \| \| | | | |

Supplementary Table 21. Multivariate Cox model adjusted with all covariates for 1-year mortality of the Non-AKI and Mild-AKI cohorts

| **Characteristic** | **HR^1^** | **95% CI^1^** | **p-value** |
| --- | --- | --- | --- |
| Group |  |  |  |
| Non-AKI | 1.00 | Reference |  |
| Mild-AKI | 0.99 | 0.81, 1.19 | 0.879 |
| Age | 1.04 | 1.04, 1.05 | <0.001 |
| Gender |  |  |  |
| Female | 1.00 | Reference |  |
| Male | 1.03 | 0.85, 1.24 | 0.799 |
| SOFA_Score (time dependent) | 1.03 | 1.02, 1.04 | <0.001 |
| Coronary_Artery_Bypass_Grafting |  |  |  |
| YES | 1.00 | Reference |  |
| NO | 6.48 | 3.20, 13.10 | <0.001 |
| Percutaneous_Coronary_Intervention |  |  |  |
| YES | 1.00 | Reference |  |
| NO | 1.17 | 0.83, 1.67 | 0.373 |
| Continuous_Renal_Replacement_Therapy |  |  |  |
| YES | 1.00 | Reference |  |
| NO | 0.90 | 0.47, 1.74 | 0.756 |
| IABP |  |  |  |
| YES | 1.00 | Reference |  |
| NO | 0.50 | 0.34, 0.72 | <0.001 |
| ACEIARB (time dependent) |  |  |  |
| YES | 1.00 | Reference |  |
| NO | 1.04 | 0.98, 1.10 | 0.174 |
| Anticoagulant (time dependent) |  |  |  |
| YES | 1.00 | Reference |  |
| NO | 0.92 | 0.86, 0.98 | 0.015 |
| Antiplatelet (time dependent) |  |  |  |
| YES | 1.00 | Reference |  |
| NO | 1.09 | 1.03, 1.16 | 0.002 |
| B_blocker (time dependent) |  |  |  |
| YES | 1.00 | Reference |  |
| NO | 1.08 | 1.02, 1.13 | 0.005 |
| Loop_diuretic (time dependent) |  |  |  |
| YES | 1.00 | Reference |  |
| NO | 0.99 | 0.94, 1.04 | 0.622 |
| Positive_inotropic |  |  |  |
| YES | 1.00 | Reference |  |
| NO | 1.33 | 0.94, 1.88 | 0.109 |
| Spironolactone |  |  |  |
| YES | 1.00 | Reference |  |
| NO | 0.94 | 0.52, 1.70 | 0.842 |
| Statin (time dependent) |  |  |  |
| YES | 1.00 | Reference |  |
| NO | 1.08 | 1.02, 1.14 | 0.005 |
| Vasopressor (time dependent) |  |  |  |
| YES | 1.00 | Reference |  |
| NO | 1.03 | 0.98, 1.10 | 0.266 |
| HF (time dependent) |  |  |  |
| YES | 1.00 | Reference |  |
| NO | 0.95 | 0.90, 1.00 | 0.046 |
| AFIB |  |  |  |
| YES | 1.00 | Reference |  |
| NO | 1.20 | 0.88, 1.64 | 0.26 |
| Diabetes (time dependent) |  |  |  |
| YES | 1.00 | Reference |  |
| NO | 0.96 | 0.92, 1.01 | 0.119 |
| Renal (time dependent) |  |  |  |
| YES | 1.00 | Reference |  |
| NO | 0.98 | 0.93, 1.03 | 0.451 |
| Liver |  |  |  |
| YES | 1.00 | Reference |  |
| NO | 0.67 | 0.31, 1.45 | 0.311 |
| COPD |  |  |  |
| YES | 1.00 | Reference |  |
| NO | 0.97 | 0.76, 1.24 | 0.811 |
| Stroke |  |  |  |
| YES | 1.00 | Reference |  |
| NO | 0.41 | 0.31, 0.54 | <0.001 |
| Malignancy (time dependent) |  |  |  |
| YES | 1.00 | Reference |  |
| NO | 0.88 | 0.83, 0.92 | <0.001 |
| MAP (time dependent) | 1.00 | 1.00, 1.00 | 0.012 |
| Heart_Rate (time dependent) | 1.00 | 1.00, 1.00 | 0.002 |
| Temperature (time dependent) | 0.94 | 0.91, 0.96 | <0.001 |
| WBC (time dependent) | 1.00 | 1.00, 1.00 | 0.508 |
| Hemoglobin (time dependent) | 0.99 | 0.98, 1.00 | 0.01 |
| Platelet | 1.00 | 1.00, 1.00 | 0.037 |
| Sodium | 1.07 | 1.04, 1.10 | <0.001 |
| Potassium | 1.07 | 0.94, 1.21 | 0.314 |
| Bicarbonate (time dependent) | 0.99 | 0.98, 1.00 | <0.001 |
| Chloride | 0.95 | 0.94, 0.97 | <0.001 |
| BUN | 1.00 | 0.99, 1.00 | 0.264 |
| eGFR | 1.00 | 0.99, 1.00 | 0.056 |
| BNP_tag |  |  |  |
| YES | 1.00 | Reference |  |
| NO | 0.97 | 0.66, 1.43 | 0.882 |
| TNT_tag (time dependent) |  |  |  |
| YES | 1.00 | Reference |  |
| NO | 0.97 | 0.91, 1.03 | 0.283 |
| CK_tag |  |  |  |
| YES | 1.00 | Reference |  |
| NO | 1.04 | 0.84, 1.28 | 0.745 |
| ^1^HR = Hazard Ratio, CI = Confidence Interval | | | |

Supplementary Table 22. Multivariate Cox model adjusted with all covariates for 1-year mortality of the Normal-or-mild-AKI and Moderate-to-severe-AKI cohorts

| **Characteristic** | **HR^1^** | **95% CI^1^** | **p-value** |
| --- | --- | --- | --- |
| Group |  |  |  |
| Normal-or-mild-AKI | 1.00 | Reference |  |
| Moderate-to-severe-AKI | 1.37 | 1.22, 1.54 | <0.001 |
| Age (time dependent) | 1.01 | 1.01, 1.01 | <0.001 |
| Gender |  |  |  |
| Female | 1.00 | Reference |  |
| Male | 0.92 | 0.83, 1.03 | 0.147 |
| SOFA_Score (time dependent) | 1.02 | 1.01, 1.02 | <0.001 |
| Coronary_Artery_Bypass_Grafting (time dependent) |  |  |  |
| YES | 1.00 | Reference |  |
| NO | 1.29 | 1.20, 1.38 | <0.001 |
| Percutaneous_Coronary_Intervention (time dependent) |  |  |  |
| YES | 1.00 | Reference |  |
| NO | 1.01 | 0.96, 1.06 | 0.787 |
| Continuous_Renal_Replacement_Therapy |  |  |  |
| YES | 1.00 | Reference |  |
| NO | 0.74 | 0.59, 0.94 | 0.012 |
| IABP (time dependent) |  |  |  |
| YES | 1.00 | Reference |  |
| NO | 0.97 | 0.93, 1.01 | 0.13 |
| ACEIARB (time dependent) |  |  |  |
| YES | 1.00 | Reference |  |
| NO | 1.08 | 1.04, 1.11 | <0.001 |
| Anticoagulant (time dependent) |  |  |  |
| YES | 1.00 | Reference |  |
| NO | 0.96 | 0.92, 1.01 | 0.082 |
| Antiplatelet (time dependent) |  |  |  |
| YES | 1.00 | Reference |  |
| NO | 1.01 | 0.98, 1.05 | 0.487 |
| B_blocker (time dependent) |  |  |  |
| YES | 1.00 | Reference |  |
| NO | 1.13 | 1.10, 1.17 | <0.001 |
| Loop_diuretic (time dependent) |  |  |  |
| YES | 1.00 | Reference |  |
| NO | 1.00 | 0.97, 1.03 | 0.959 |
| Positive_inotropic |  |  |  |
| YES | 1.00 | Reference |  |
| NO | 0.90 | 0.78, 1.04 | 0.168 |
| Spironolactone |  |  |  |
| YES | 1.00 | Reference |  |
| NO | 0.69 | 0.51, 0.93 | 0.015 |
| Statin (time dependent) |  |  |  |
| YES | 1.00 | Reference |  |
| NO | 1.06 | 1.02, 1.09 | 0.002 |
| Vasopressor (time dependent) |  |  |  |
| YES | 1.00 | Reference |  |
| NO | 0.96 | 0.93, 0.99 | 0.022 |
| HF (time dependent) |  |  |  |
| YES | 1.00 | Reference |  |
| NO | 0.93 | 0.91, 0.96 | <0.001 |
| AFIB |  |  |  |
| YES | 1.00 | Reference |  |
| NO | 0.98 | 0.84, 1.13 | 0.749 |
| Diabetes (time dependent) |  |  |  |
| YES | 1.00 | Reference |  |
| NO | 0.97 | 0.95, 1.00 | 0.034 |
| Renal_Disease (time dependent) |  |  |  |
| YES | 1.00 | Reference |  |
| NO | 1.03 | 1.00, 1.06 | 0.097 |
| Liver_Disease |  |  |  |
| YES | 1.00 | Reference |  |
| NO | 0.63 | 0.45, 0.88 | 0.006 |
| COPD |  |  |  |
| YES | 1.00 | Reference |  |
| NO | 0.77 | 0.68, 0.87 | <0.001 |
| Stroke |  |  |  |
| YES | 1.00 | Reference |  |
| NO | 0.60 | 0.52, 0.69 | <0.001 |
| Malignancy (time dependent) |  |  |  |
| YES | 1.00 | Reference |  |
| NO | 0.91 | 0.88, 0.94 | <0.001 |
| MAP (time dependent) | 1.00 | 1.00, 1.00 | 0.772 |
| Heart_Rate (time dependent) | 1.00 | 1.00, 1.00 | <0.001 |
| Temperature (time dependent) | 0.97 | 0.95, 0.98 | <0.001 |
| WBC (time dependent) | 1.00 | 1.00, 1.00 | 0.556 |
| Hemoglobin (time dependent) | 0.99 | 0.98, 1.00 | <0.001 |
| Platelet | 1.00 | 1.00, 1.00 | <0.001 |
| Sodium | 1.04 | 1.03, 1.05 | <0.001 |
| Potassium | 1.01 | 0.95, 1.09 | 0.699 |
| Bicarbonate (time dependent) | 1.00 | 0.99, 1.00 | 0.003 |
| Chloride | 0.96 | 0.95, 0.97 | <0.001 |
| BUN | 1.00 | 1.00, 1.01 | 0.009 |
| eGFR | 0.99 | 0.99, 1.00 | <0.001 |
| BNP_tag |  |  |  |
| YES | 1.00 | Reference |  |
| NO | 1.08 | 0.87, 1.32 | 0.491 |
| TNT_tag |  |  |  |
| YES | 1.00 | Reference |  |
| NO | 0.88 | 0.76, 1.01 | 0.078 |
| CK_tag |  |  |  |
| YES | 1.00 | Reference |  |
| NO | 0.88 | 0.79, 1.00 | 0.043 |
| ^1^HR = Hazard Ratio, CI = Confidence Interval | | | |

Supplementary Table 23. Multivariate Cox model adjusted with unbalanced covariates and IPTW for 1-year mortality of the Non-AKI and Mild-AKI cohorts

| **Characteristic** | **HR^1^** | **95% CI^1^** | **p-value** |
| --- | --- | --- | --- |
| Group |  |  |  |
| Non-AKI | 1.00 | Reference |  |
| Mild-AKI | 1.05 | 0.87, 1.27 | 0.626 |
| Age (time dependent) | 1.01 | 1.01, 1.01 | <0.001 |
| SOFA_Score (time dependent) | 1.04 | 1.03, 1.05 | <0.001 |
| Coronary_Artery_Bypass_Grafting |  |  |  |
| YES | 1.00 | Reference |  |
| NO | 7.10 | 3.56, 14.18 | <0.001 |
| Percutaneous_Coronary_Intervention |  |  |  |
| YES | 1.00 | Reference |  |
| NO | 1.36 | 0.96, 1.92 | 0.081 |
| Continuous_Renal_Replacement_Therapy |  |  |  |
| YES | 1.00 | Reference |  |
| NO | 0.64 | 0.35, 1.19 | 0.16 |
| IABP |  |  |  |
| YES | 1.00 | Reference |  |
| NO | 0.57 | 0.39, 0.82 | 0.003 |
| B_blocker (time dependent) |  |  |  |
| YES | 1.00 | Reference |  |
| NO | 1.10 | 1.05, 1.16 | <0.001 |
| Loop_diuretic (time dependent) |  |  |  |
| YES | 1.00 | Reference |  |
| NO | 1.00 | 0.96, 1.05 | 0.903 |
| Positive_inotropic |  |  |  |
| YES | 1.00 | Reference |  |
| NO | 1.55 | 1.10, 2.18 | 0.011 |
| Statin (time dependent) |  |  |  |
| YES | 1.00 | Reference |  |
| NO | 1.11 | 1.05, 1.16 | <0.001 |
| Vasopressor (time dependent) |  |  |  |
| YES | 1.00 | Reference |  |
| NO | 1.04 | 0.98, 1.10 | 0.2 |
| HF (time dependent) |  |  |  |
| YES | 1.00 | Reference |  |
| NO | 0.94 | 0.89, 0.98 | 0.008 |
| Diabetes (time dependent) |  |  |  |
| YES | 1.00 | Reference |  |
| NO | 0.96 | 0.92, 1.00 | 0.059 |
| Renal (time dependent) |  |  |  |
| YES | 1.00 | Reference |  |
| NO | 0.99 | 0.94, 1.04 | 0.656 |
| MAP (time dependent) | 1.00 | 1.00, 1.00 | 0.335 |
| Hemoglobin (time dependent) | 0.99 | 0.98, 1.00 | 0.138 |
| Platelet | 1.00 | 1.00, 1.00 | 0.001 |
| Sodium | 1.02 | 1.00, 1.04 | 0.03 |
| Potassium | 1.03 | 0.90, 1.17 | 0.704 |
| Bicarbonate (time dependent) | 0.99 | 0.99, 1.00 | 0.002 |
| BUN | 1.00 | 0.99, 1.00 | 0.814 |
| eGFR | 0.99 | 0.99, 1.00 | 0.027 |
| TNT_tag (time dependent) |  |  |  |
| YES | 1.00 | Reference |  |
| NO | 0.95 | 0.89, 1.00 | 0.066 |
| CK_tag |  |  |  |
| YES | 1.00 | Reference |  |
| NO | 1.04 | 0.85, 1.27 | 0.708 |
| ^1^HR = Hazard Ratio, CI = Confidence Interval  Supplementary Table 24. Multivariate Cox model adjusted with unbalanced covariates and IPTW for 1-year mortality of the Normal-or-mild-AKI and Moderate-to-severe-AKI cohorts   \| **Characteristic** \| **HR^1^** \| **95% CI^1^** \| **p-value** \| \| --- \| --- \| --- \| --- \| \| Group \|  \|  \|  \| \| Normal-or-mild-AKI \| 1.00 \| Reference \|  \| \| Moderate-to-severe-AKI \| 1.28 \| 1.14, 1.44 \| <0.001 \| \| Age (time dependent) \| 1.01 \| 1.01, 1.01 \| <0.001 \| \| SOFA_Score (time dependent) \| 1.02 \| 1.02, 1.03 \| <0.001 \| \| Percutaneous_Coronary_Intervention (time dependent) \|  \|  \|  \| \| YES \| 1.00 \| Reference \|  \| \| NO \| 1.01 \| 0.97, 1.07 \| 0.57 \| \| Continuous_Renal_Replacement_Therapy \|  \|  \|  \| \| YES \| 1.00 \| Reference \|  \| \| NO \| 0.73 \| 0.58, 0.91 \| 0.006 \| \| IABP (time dependent) \|  \|  \|  \| \| YES \| 1.00 \| Reference \|  \| \| NO \| 1.00 \| 0.96, 1.04 \| 0.884 \| \| Anticoagulant (time dependent) \|  \|  \|  \| \| YES \| 1.00 \| Reference \|  \| \| NO \| 0.96 \| 0.92, 1.00 \| 0.066 \| \| Loop_diuretic (time dependent) \|  \|  \|  \| \| YES \| 1.00 \| Reference \|  \| \| NO \| 1.03 \| 1.00, 1.06 \| 0.029 \| \| Statin (time dependent) \|  \|  \|  \| \| YES \| 1.00 \| Reference \|  \| \| NO \| 1.13 \| 1.09, 1.16 \| <0.001 \| \| Vasopressor (time dependent) \|  \|  \|  \| \| YES \| 1.00 \| Reference \|  \| \| NO \| 0.98 \| 0.95, 1.01 \| 0.197 \| \| HF (time dependent) \|  \|  \|  \| \| YES \| 1.00 \| Reference \|  \| \| NO \| 0.92 \| 0.90, 0.95 \| <0.001 \| \| AFIB \|  \|  \|  \| \| YES \| 1.00 \| Reference \|  \| \| NO \| 0.95 \| 0.83, 1.10 \| 0.506 \| \| Diabetes (time dependent) \|  \|  \|  \| \| YES \| 1.00 \| Reference \|  \| \| NO \| 0.98 \| 0.95, 1.00 \| 0.08 \| \| Renal_Disease (time dependent) \|  \|  \|  \| \| YES \| 1.00 \| Reference \|  \| \| NO \| 1.04 \| 1.01, 1.07 \| 0.022 \| \| Stroke \|  \|  \|  \| \| YES \| 1.00 \| Reference \|  \| \| NO \| 0.60 \| 0.52, 0.70 \| <0.001 \| \| Heart_Rate (time dependent) \| 1.00 \| 1.00, 1.00 \| <0.001 \| \| WBC (time dependent) \| 1.00 \| 1.00, 1.00 \| 0.791 \| \| Bicarbonate (time dependent) \| 1.00 \| 0.99, 1.00 \| 0.007 \| \| Chloride \| 0.98 \| 0.97, 0.99 \| <0.001 \| \| BUN \| 1.01 \| 1.00, 1.01 \| <0.001 \| \| eGFR \| 0.99 \| 0.99, 1.00 \| <0.001 \| \| TNT_tag (time dependent) \|  \|  \|  \| \| YES \| 1.00 \| Reference \|  \| \| NO \| 0.91 \| 0.87, 0.94 \| <0.001 \| \| CK_tag \|  \|  \|  \| \| YES \| 1.00 \| Reference \|  \| \| NO \| 0.92 \| 0.82, 1.03 \| 0.164 \| \| ^1^HR = Hazard Ratio, CI = Confidence Interval \| \| \| \| | | | |

Supplementary Table 25. Multivariate Cox model adjusted with all covariates and IPTW for 1-year mortality of the Non-AKI and Mild-AKI cohorts

| **Characteristic** | **HR^1^** | **95% CI^1^** | **p-value** |
| --- | --- | --- | --- |
| Group |  |  |  |
| Non-AKI | 1.00 | Reference |  |
| Mild-AKI | 0.93 | 0.76, 1.14 | 0.484 |
| Age (time dependent) | 1.01 | 1.01, 1.01 | <0.001 |
| Gender |  |  |  |
| Female | 1.00 | Reference |  |
| Male | 1.00 | 0.81, 1.23 | 0.991 |
| SOFA_Score (time dependent) | 1.03 | 1.02, 1.04 | <0.001 |
| Coronary_Artery_Bypass_Grafting |  |  |  |
| YES | 1.00 | Reference |  |
| NO | 5.61 | 2.61, 12.06 | <0.001 |
| Percutaneous_Coronary_Intervention |  |  |  |
| YES | 1.00 | Reference |  |
| NO | 1.13 | 0.78, 1.65 | 0.513 |
| Continuous_Renal_Replacement_Therapy (time dependent) |  |  |  |
| YES | 1.00 | Reference |  |
| NO | 0.94 | 0.78, 1.13 | 0.497 |
| IABP |  |  |  |
| YES | 1.00 | Reference |  |
| NO | 0.53 | 0.35, 0.79 | 0.002 |
| ACEIARB (time dependent) |  |  |  |
| YES | 1.00 | Reference |  |
| NO | 1.04 | 0.97, 1.11 | 0.269 |
| Anticoagulant (time dependent) |  |  |  |
| YES | 1.00 | Reference |  |
| NO | 0.90 | 0.84, 0.97 | 0.007 |
| Antiplatelet (time dependent) |  |  |  |
| YES | 1.00 | Reference |  |
| NO | 1.10 | 1.03, 1.18 | 0.004 |
| B_blocker (time dependent) |  |  |  |
| YES | 1.00 | Reference |  |
| NO | 1.06 | 1.00, 1.12 | 0.045 |
| Loop_diuretic (time dependent) |  |  |  |
| YES | 1.00 | Reference |  |
| NO | 0.97 | 0.92, 1.02 | 0.248 |
| Positive_inotropic |  |  |  |
| YES | 1.00 | Reference |  |
| NO | 1.32 | 0.91, 1.93 | 0.148 |
| Spironolactone |  |  |  |
| YES | 1.00 | Reference |  |
| NO | 0.86 | 0.46, 1.60 | 0.639 |
| Statin (time dependent) |  |  |  |
| YES | 1.00 | Reference |  |
| NO | 1.08 | 1.02, 1.14 | 0.007 |
| Vasopressor (time dependent) |  |  |  |
| YES | 1.00 | Reference |  |
| NO | 1.04 | 0.97, 1.11 | 0.28 |
| HF (time dependent) |  |  |  |
| YES | 1.00 | Reference |  |
| NO | 0.94 | 0.90, 0.99 | 0.029 |
| AFIB |  |  |  |
| YES | 1.00 | Reference |  |
| NO | 1.27 | 0.91, 1.79 | 0.159 |
| Diabetes (time dependent) |  |  |  |
| YES | 1.00 | Reference |  |
| NO | 0.97 | 0.92, 1.02 | 0.204 |
| Renal (time dependent) |  |  |  |
| YES | 1.00 | Reference |  |
| NO | 0.98 | 0.92, 1.05 | 0.61 |
| Liver |  |  |  |
| YES | 1.00 | Reference |  |
| NO | 0.74 | 0.33, 1.67 | 0.476 |
| COPD |  |  |  |
| YES | 1.00 | Reference |  |
| NO | 0.96 | 0.73, 1.26 | 0.782 |
| Stroke |  |  |  |
| YES | 1.00 | Reference |  |
| NO | 0.41 | 0.30, 0.56 | <0.001 |
| Malignancy (time dependent) |  |  |  |
| YES | 1.00 | Reference |  |
| NO | 0.87 | 0.82, 0.93 | <0.001 |
| MAP (time dependent) | 1.00 | 1.00, 1.00 | 0.093 |
| Heart_Rate (time dependent) | 1.00 | 1.00, 1.00 | 0.005 |
| Temperature (time dependent) | 0.94 | 0.91, 0.97 | <0.001 |
| WBC (time dependent) | 1.00 | 1.00, 1.00 | 0.617 |
| Hemoglobin (time dependent) | 0.99 | 0.98, 1.00 | 0.041 |
| Platelet | 1.00 | 1.00, 1.00 | 0.053 |
| Sodium | 1.07 | 1.04, 1.10 | <0.001 |
| Potassium | 1.05 | 0.91, 1.22 | 0.485 |
| Bicarbonate (time dependent) | 0.99 | 0.98, 1.00 | 0.004 |
| Chloride (time dependent) | 0.99 | 0.99, 0.99 | <0.001 |
| BUN | 1.00 | 0.99, 1.00 | 0.283 |
| eGFR | 0.99 | 0.99, 1.00 | 0.082 |
| BNP_tag |  |  |  |
| YES | 1.00 | Reference |  |
| NO | 0.97 | 0.65, 1.45 | 0.877 |
| TNT_tag (time dependent) |  |  |  |
| YES | 1.00 | Reference |  |
| NO | 0.97 | 0.90, 1.04 | 0.395 |
| CK_tag |  |  |  |
| YES | 1.00 | Reference |  |
| NO | 1.04 | 0.83, 1.31 | 0.707 |
| ^1^HR = Hazard Ratio, CI = Confidence Interval  Supplementary Table 26. Survey-weighted Cox model adjusted with all covariates and IPTW for 1-year mortality of the Normal-or-mild-AKI and Moderate-to-severe-AKI cohorts   \| **Characteristic** \| **HR^1^** \| **95% CI^1^** \| **p-value** \| \| --- \| --- \| --- \| --- \| \| Group \|  \|  \|  \| \| Normal-or-mild-AKI \| 1.00 \| Reference \|  \| \| Moderate-to-severe-AKI \| 1.26 \| 1.11, 1.43 \| <0.001 \| \| Age (time dependent) \| 1.01 \| 1.01, 1.01 \| <0.001 \| \| Gender \|  \|  \|  \| \| Female \| 1.00 \| Reference \|  \| \| Male \| 0.89 \| 0.79, 1.01 \| 0.067 \| \| SOFA_Score (time dependent) \| 1.02 \| 1.02, 1.03 \| <0.001 \| \| Coronary_Artery_Bypass_Grafting (time dependent) \|  \|  \|  \| \| YES \| 1.00 \| Reference \|  \| \| NO \| 1.28 \| 1.18, 1.39 \| <0.001 \| \| Percutaneous_Coronary_Intervention (time dependent) \|  \|  \|  \| \| YES \| 1.00 \| Reference \|  \| \| NO \| 1.01 \| 0.95, 1.06 \| 0.84 \| \| Continuous_Renal_Replacement_Therapy \|  \|  \|  \| \| YES \| 1.00 \| Reference \|  \| \| NO \| 0.71 \| 0.54, 0.94 \| 0.015 \| \| IABP (time dependent) \|  \|  \|  \| \| YES \| 1.00 \| Reference \|  \| \| NO \| 0.95 \| 0.90, 1.00 \| 0.053 \| \| ACEIARB (time dependent) \|  \|  \|  \| \| YES \| 1.00 \| Reference \|  \| \| NO \| 1.07 \| 1.03, 1.11 \| <0.001 \| \| Anticoagulant (time dependent) \|  \|  \|  \| \| YES \| 1.00 \| Reference \|  \| \| NO \| 0.97 \| 0.92, 1.02 \| 0.256 \| \| Antiplatelet (time dependent) \|  \|  \|  \| \| YES \| 1.00 \| Reference \|  \| \| NO \| 1.02 \| 0.98, 1.06 \| 0.367 \| \| B_blocker (time dependent) \|  \|  \|  \| \| YES \| 1.00 \| Reference \|  \| \| NO \| 1.12 \| 1.08, 1.15 \| <0.001 \| \| Loop_diuretic (time dependent) \|  \|  \|  \| \| YES \| 1.00 \| Reference \|  \| \| NO \| 1.00 \| 0.97, 1.03 \| 0.811 \| \| Positive_inotropic \|  \|  \|  \| \| YES \| 1.00 \| Reference \|  \| \| NO \| 0.88 \| 0.74, 1.04 \| 0.142 \| \| Spironolactone \|  \|  \|  \| \| YES \| 1.00 \| Reference \|  \| \| NO \| 0.69 \| 0.50, 0.96 \| 0.027 \| \| Statin (time dependent) \|  \|  \|  \| \| YES \| 1.00 \| Reference \|  \| \| NO \| 1.06 \| 1.02, 1.10 \| 0.001 \| \| Vasopressor (time dependent) \|  \|  \|  \| \| YES \| 1.00 \| Reference \|  \| \| NO \| 0.97 \| 0.94, 1.01 \| 0.095 \| \| HF (time dependent) \|  \|  \|  \| \| YES \| 1.00 \| Reference \|  \| \| NO \| 0.94 \| 0.91, 0.97 \| <0.001 \| \| AFIB \|  \|  \|  \| \| YES \| 1.00 \| Reference \|  \| \| NO \| 1.05 \| 0.89, 1.24 \| 0.559 \| \| Diabetes (time dependent) \|  \|  \|  \| \| YES \| 1.00 \| Reference \|  \| \| NO \| 0.97 \| 0.94, 1.00 \| 0.024 \| \| Renal_Disease (time dependent) \|  \|  \|  \| \| YES \| 1.00 \| Reference \|  \| \| NO \| 1.01 \| 0.97, 1.05 \| 0.687 \| \| Liver_Disease \|  \|  \|  \| \| YES \| 1.00 \| Reference \|  \| \| NO \| 0.52 \| 0.36, 0.75 \| <0.001 \| \| COPD \|  \|  \|  \| \| YES \| 1.00 \| Reference \|  \| \| NO \| 0.79 \| 0.68, 0.91 \| 0.001 \| \| Stroke \|  \|  \|  \| \| YES \| 1.00 \| Reference \|  \| \| NO \| 0.55 \| 0.46, 0.64 \| <0.001 \| \| Malignancy (time dependent) \|  \|  \|  \| \| YES \| 1.00 \| Reference \|  \| \| NO \| 0.90 \| 0.87, 0.94 \| <0.001 \| \| MAP (time dependent) \| 1.00 \| 1.00, 1.00 \| 0.604 \| \| Heart_Rate (time dependent) \| 1.00 \| 1.00, 1.00 \| <0.001 \| \| Temperature (time dependent) \| 0.97 \| 0.96, 0.99 \| 0.001 \| \| WBC (time dependent) \| 1.00 \| 1.00, 1.00 \| 0.372 \| \| Hemoglobin (time dependent) \| 0.99 \| 0.98, 1.00 \| 0.005 \| \| Platelet \| 1.00 \| 1.00, 1.00 \| <0.001 \| \| Sodium \| 1.04 \| 1.03, 1.05 \| <0.001 \| \| Potassium \| 1.00 \| 0.92, 1.09 \| 0.98 \| \| Bicarbonate (time dependent) \| 0.99 \| 0.99, 1.00 \| 0.001 \| \| Chloride \| 0.96 \| 0.95, 0.97 \| <0.001 \| \| BUN \| 1.00 \| 1.00, 1.01 \| 0.03 \| \| eGFR \| 1.00 \| 0.99, 1.00 \| 0.013 \| \| BNP_tag \|  \|  \|  \| \| YES \| 1.00 \| Reference \|  \| \| NO \| 1.07 \| 0.84, 1.36 \| 0.583 \| \| TNT_tag \|  \|  \|  \| \| YES \| 1.00 \| Reference \|  \| \| NO \| 0.83 \| 0.70, 0.97 \| 0.022 \| \| CK_tag \|  \|  \|  \| \| YES \| 1.00 \| Reference \|  \| \| NO \| 0.90 \| 0.79, 1.02 \| 0.105 \| \| ^1^HR = Hazard Ratio, CI = Confidence Interval \| \| \| \| | | | |

Supplementary Table 27. Survey-weighted Cox model adjusted with unbalanced covariates and IPTW for 1-year mortality of the Non-AKI and Mild-AKI cohorts

| **Characteristic** | **HR^1^** | **95% CI^1^** | **p-value** |
| --- | --- | --- | --- |
| Group |  |  |  |
| Non-AKI | 1.00 | Reference |  |
| Mild-AKI | 0.98 | 0.81, 1.20 | 0.857 |
| Age (time dependent) | 1.01 | 1.01, 1.01 | <0.001 |
| SOFA_Score (time dependent) | 1.04 | 1.03, 1.05 | <0.001 |
| Coronary_Artery_Bypass_Grafting |  |  |  |
| YES | 1.00 | Reference |  |
| NO | 6.16 | 2.93, 12.98 | <0.001 |
| Percutaneous_Coronary_Intervention |  |  |  |
| YES | 1.00 | Reference |  |
| NO | 1.30 | 0.90, 1.88 | 0.168 |
| Continuous_Renal_Replacement_Therapy |  |  |  |
| YES | 1.00 | Reference |  |
| NO | 0.64 | 0.33, 1.21 | 0.17 |
| IABP |  |  |  |
| YES | 1.00 | Reference |  |
| NO | 0.60 | 0.40, 0.90 | 0.013 |
| B_blocker (time dependent) |  |  |  |
| YES | 1.00 | Reference |  |
| NO | 1.09 | 1.04, 1.14 | <0.001 |
| Loop_diuretic (time dependent) |  |  |  |
| YES | 1.00 | Reference |  |
| NO | 0.99 | 0.94, 1.05 | 0.776 |
| Positive_inotropic |  |  |  |
| YES | 1.00 | Reference |  |
| NO | 1.50 | 1.04, 2.17 | 0.029 |
| Statin (time dependent) |  |  |  |
| YES | 1.00 | Reference |  |
| NO | 1.11 | 1.05, 1.16 | <0.001 |
| Vasopressor (time dependent) |  |  |  |
| YES | 1.00 | Reference |  |
| NO | 1.04 | 0.98, 1.12 | 0.214 |
| HF (time dependent) |  |  |  |
| YES | 1.00 | Reference |  |
| NO | 0.92 | 0.88, 0.97 | 0.001 |
| Diabetes (time dependent) |  |  |  |
| YES | 1.00 | Reference |  |
| NO | 0.96 | 0.91, 1.00 | 0.056 |
| Renal (time dependent) |  |  |  |
| YES | 1.00 | Reference |  |
| NO | 0.98 | 0.93, 1.04 | 0.598 |
| MAP (time dependent) | 1.00 | 1.00, 1.00 | 0.619 |
| Hemoglobin (time dependent) | 0.99 | 0.98, 1.01 | 0.376 |
| Platelet | 1.00 | 1.00, 1.00 | 0.003 |
| Sodium | 1.03 | 1.00, 1.05 | 0.057 |
| Potassium | 1.01 | 0.88, 1.18 | 0.853 |
| Bicarbonate (time dependent) | 0.99 | 0.98, 1.00 | 0.012 |
| BUN | 1.00 | 0.99, 1.00 | 0.675 |
| eGFR | 0.99 | 0.99, 1.00 | 0.097 |
| TNT_tag (time dependent) |  |  |  |
| YES | 1.00 | Reference |  |
| NO | 0.95 | 0.89, 1.02 | 0.144 |
| CK_tag |  |  |  |
| YES | 1.00 | Reference |  |
| NO | 1.03 | 0.84, 1.28 | 0.767 |
| ^1^HR = Hazard Ratio, CI = Confidence Interval  Supplementary Table 28. Survey-weighted Cox model adjusted with unbalanced covariates and IPTW for 1-year mortality of the Normal-or-mild-AKI and Moderate-to-severe-AKI cohorts   \| **Characteristic** \| **HR^1^** \| **95% CI^1^** \| **p-value** \| \| --- \| --- \| --- \| --- \| \| Group \|  \|  \|  \| \| Normal-or-mild-AKI \| 1.00 \| Reference \|  \| \| Moderate-to-severe-AKI \| 1.22 \| 1.07, 1.38 \| 0.002 \| \| Age (time dependent) \| 1.01 \| 1.01, 1.01 \| <0.001 \| \| SOFA_Score (time dependent) \| 1.02 \| 1.02, 1.03 \| <0.001 \| \| Percutaneous_Coronary_Intervention (time dependent) \|  \|  \|  \| \| YES \| 1.00 \| Reference \|  \| \| NO \| 1.01 \| 0.96, 1.07 \| 0.613 \| \| Continuous_Renal_Replacement_Therapy \|  \|  \|  \| \| YES \| 1.00 \| Reference \|  \| \| NO \| 0.71 \| 0.54, 0.93 \| 0.014 \| \| IABP (time dependent) \|  \|  \|  \| \| YES \| 1.00 \| Reference \|  \| \| NO \| 0.97 \| 0.93, 1.02 \| 0.302 \| \| Anticoagulant (time dependent) \|  \|  \|  \| \| YES \| 1.00 \| Reference \|  \| \| NO \| 0.97 \| 0.92, 1.02 \| 0.192 \| \| Loop_diuretic (time dependent) \|  \|  \|  \| \| YES \| 1.00 \| Reference \|  \| \| NO \| 1.03 \| 1.00, 1.06 \| 0.054 \| \| Statin (time dependent) \|  \|  \|  \| \| YES \| 1.00 \| Reference \|  \| \| NO \| 1.13 \| 1.09, 1.16 \| <0.001 \| \| Vasopressor (time dependent) \|  \|  \|  \| \| YES \| 1.00 \| Reference \|  \| \| NO \| 0.99 \| 0.95, 1.02 \| 0.495 \| \| HF (time dependent) \|  \|  \|  \| \| YES \| 1.00 \| Reference \|  \| \| NO \| 0.92 \| 0.90, 0.95 \| <0.001 \| \| AFIB \|  \|  \|  \| \| YES \| 1.00 \| Reference \|  \| \| NO \| 1.05 \| 0.89, 1.23 \| 0.569 \| \| Diabetes (time dependent) \|  \|  \|  \| \| YES \| 1.00 \| Reference \|  \| \| NO \| 0.97 \| 0.94, 1.00 \| 0.055 \| \| Renal_Disease (time dependent) \|  \|  \|  \| \| YES \| 1.00 \| Reference \|  \| \| NO \| 1.02 \| 0.98, 1.06 \| 0.315 \| \| Stroke \|  \|  \|  \| \| YES \| 1.00 \| Reference \|  \| \| NO \| 0.54 \| 0.46, 0.64 \| <0.001 \| \| Heart_Rate (time dependent) \| 1.00 \| 1.00, 1.00 \| <0.001 \| \| WBC (time dependent) \| 1.00 \| 1.00, 1.00 \| 0.578 \| \| Bicarbonate (time dependent) \| 0.99 \| 0.99, 1.00 \| 0.005 \| \| Chloride \| 0.97 \| 0.96, 0.98 \| <0.001 \| \| BUN \| 1.01 \| 1.00, 1.01 \| <0.001 \| \| eGFR \| 0.99 \| 0.99, 1.00 \| 0.002 \| \| TNT_tag (time dependent) \|  \|  \|  \| \| YES \| 1.00 \| Reference \|  \| \| NO \| 0.90 \| 0.86, 0.93 \| <0.001 \| \| CK_tag \|  \|  \|  \| \| YES \| 1.00 \| Reference \|  \| \| NO \| 0.92 \| 0.81, 1.05 \| 0.208 \| \| ^1^HR = Hazard Ratio, CI = Confidence Interval \| \| \| \| | | | |
